# Coupling Coordination Degree Evaluation of Regional Water Resources, Social Economy and Ecological Environment Composite System Based on Logical Multiplication of Connection Numbers and Stochastic Simulations

**DOI:** 10.3390/e28090982

**Published:** 2026-09-02

**Authors:** Ziyu Wang, Juliang Jin, Liangguang Zhou, Rongxing Zhou, Chengguo Wu, Yi Cui, Yawei Wang, Chao Huang

**Affiliations:** 1 College of Civil Engineering, Hefei University of Technology, Hefei 230009, China; hfutwangziyu@163.com (Z.W.); jinjl66@126.com (J.J.); wule9825@163.com (C.W.); ycui@hfut.edu.cn (Y.C.); hfutwangyawei@163.com (Y.W.); huangchao_hfut@163.com (C.H.); 2 Joint Laboratory of Artificial Intelligence for Water Security, Hefei University of Technology, Hefei 230009, China; 3 School of Geographic Engineering and Spatial Information, Chuzhou University, Chuzhou 239000, China; 4 School of Environment and Energy Engineering, Anhui Jianzhu University, Hefei 230601, China; zhourx11@163.com

**Keywords:** evaluation of coupling coordination degree, logical multiplication of connection numbers, overall partial connection number, stochastic simulation, Jing River Basin

## Abstract

Traditional coupling coordination degree (CCD) evaluation methods fail to simultaneously ensure the accuracy and reliability of evaluation results. To overcome this limitation, a novel evaluation method that integrates the logical multiplication of connection numbers with stochastic simulations (ECCD-LMS) is developed to assess the CCD of regional water resources, social economy, and ecological environment (WSE) composite systems. The method adopts three-element connection numbers to quantify the comprehensive evaluation level of each system. Overall partial connection numbers and triangular fuzzy numbers then define dynamic value intervals for the connection number components, and the Monte Carlo method is integrated to simulate stochastic variation in each component. The simulated values are substituted into the logical multiplication of connection numbers to generate evaluation results that include both mean CCD estimates and 95% uncertainty intervals. The empirical application of ECCD-LMS in China’s Jing River Basin indicated that the coupling coordination level of the regional WSE composite system exhibited an overall increasing tendency with fluctuations from 2012 to 2023. These fluctuations were closely associated with variations in the comprehensive evaluation level of the water resources system. Spatially, the disparities in evaluation grades among subregions gradually narrowed during the study period. Compared with traditional CCD evaluation methods, ECCD-LMS effectively corrects systematic overestimation while maintaining objectivity and produces more dispersed CCD estimates that reflect differences among evaluation samples with greater clarity. Furthermore, by outputting mean CCD estimates and 95% uncertainty intervals, ECCD-LMS outperforms traditional methods that only provide static point estimates. It thus enables robust and credible evaluation of the coupling coordination level of WSE composite systems under multiple sources of uncertainty, suggesting potential applicability across diverse regional contexts.

## 1. Introduction

In regional development, intricate interdependence and mutual constraint mechanisms shape complex relationships among water resource security, socioeconomic growth, and eco-environment protection [1]. As a fundamental natural resource, water provides sustained support for both socioeconomic development systems and natural ecosystems. Conversely, high-quality socioeconomic development provides sufficient financial investment and advanced technical support for the efficient utilization of water resources and proactive environmental governance. Meanwhile, healthy ecosystems sustain water cycle processes while also supporting regional economic development [2]. Such a high degree of interconnectedness among water resources, socioeconomic development, and the ecological environment leads to the formation of a dynamically coupled composite system comprising all three individual systems [3]. However, rapid economic growth and unsustainable resource exploitation have triggered escalating conflicts among water resource conservation, socioeconomic development, and ecological environment protection, frequently resulting in functional disorders and functional dysfunctions within the WSE composite system [4]. In this context, objective evaluation of the coordinated development of the WSE composite system is critically essential for regional sustainability development and identification of obstacles within the composite system [5].

The coupling coordination degree (CCD) model is a commonly used tool for quantitatively analyzing coupling coordination relationships within complex systems [6,7]. In current practice, “evaluation method of coupling coordination degree by common way” (ECCD-CW) [2,8,9,10] derives comprehensive evaluation values for each system by summing weighted indicator sample data, typically after processing through range standardization to eliminate dimensional differences. However, these comprehensive evaluation results are specific to a particular sample dataset and also fluctuate with the maximum or minimum values of the indicator data, making comparisons among studies or systems difficult [8]. Furthermore, the coupling degree is calculated by dividing the geometric mean of the comprehensive evaluation values from all systems by their arithmetic mean [7,8,11,12]; this results in a left-skewed distribution between zero and one [13]. If all systems are assigned equal weights, the CCD model degenerates into the square root of the geometric mean of the comprehensive evaluation values, leading to inflation of CCD and a tendency to produce systematically higher CCD estimates [8]. To address this, an improved method, “evaluation method of coupling coordination degree based on connection numbers” (ECCD-CN), has since been proposed based on set pair analysis [14]. ECCD-CN uses the subtraction set pair potential of three-element connection numbers from set pair analysis to characterize the relative membership degree for each system’s comprehensive evaluation level. The geometric mean is then used to calculate the coupling degree (*C_i_*) among the systems for sample *i* across spatiotemporal scales, and the arithmetic mean is employed to calculate a comprehensive evaluation value (*T_i_*) for the composite system. Finally, the CCD (*D_i_*) of the composite system is obtained through the geometric mean (*C_i_T_i_*)^0.5^. Early applications of ECCD-CN show that it can accurately estimate CCD [8]. However, due to sampling uncertainty for evaluation indicator values in CCD estimation, as well as uncertainty in judging and calculating the proximity between indicator values and evaluation grade criteria, the comprehensive evaluation value of each system is not actually a deterministic real number [8]. As the core mathematical tool of set pair analysis, the connection number has a unique advantage in handling the relationship between certainty and uncertainty. Therefore, connection numbers can be used to directly characterize the comprehensive evaluation level of each system. On this basis, in an extension of the set pair analysis approach, the logical multiplication of pairs of three-element connection numbers was proposed as an amendment to CCD evaluation of water resource supply-side and demand-side systems [8]. This led to the development of “evaluation method of coupling coordination degree based on set pair reasoning” (ECCD-SPR), useful for assessing the carrying capacity of regional water resources. By relying on the use of a logical process to determine CCD, ECCD-SPR offers greater interpretability. ECCD-SPR has been extended to specifically assess coupling coordination relationships among water resources, social economy, and ecological environment systems [15]. This extension, termed “evaluation method of coupling coordination degree based on the logical multiplication of three connection numbers” (ECCD-MCN), was implemented in an empirical study of the Chengdu-Chongqing urban agglomeration, further advancing theoretical and applied research on set pair logical reasoning in the field of water resources. ECCD-SPR and ECCD-MCN rely on same, different, and opposite relationship information for the comprehensive evaluation of connection numbers of each system. The methods quantify interactions among systems from three perspectives, maintaining consistency in dimensional type and order-of-magnitude between CCD estimates and the comprehensive evaluation levels of the individual systems, thereby improving the rationality and interpretability of the evaluation results.

As a core input for CCD evaluation, the sameness (*a*), difference (*b*), and opposition (*c*) components of each system’s comprehensive evaluation connection number jointly constitute the sameness-difference-opposition system structure of the connection number [16]. Note that these components are not mutually independent, as they exhibit mutual migration and dynamic evolution at the microscopic level [16]. This leads to fluctuation and uncertainty in the CCD evaluation results of the composite system [16]. However, both ECCD-MCN and ECCD-SPR are deterministic, producing a single CCD value that fails to capture uncertainty caused by dynamic transformations among the connection number components. To quantify this uncertainty, dynamic value intervals may be defined for each system’s comprehensive evaluation connection number components. Partial connection numbers [17] and semi-partial connection numbers [16] capture micro-level movement between certainty (sameness/opposition) and uncertainty (difference) within the relational structure of a set pair system, measuring transformations among the connection number components. However, the focus is solely on local transformations [16,17], without accounting for the fact that when the difference component (*b*) simultaneously migrates toward both the sameness (*a*) and opposition (*c*) components, the total migration parameter may become excessively large. This can result in a connection number outside the interval [−1, 1] [16]. Overall partial connection numbers [16], under the global constraint of *a* + *b* + *c* = 1, therefore more reasonably capture the dynamics of each connection number component, providing a more rigorous mathematical basis for determining the dynamic value intervals. Given that the original values calculated for each system’s comprehensive evaluation connection number components represent the most probable values under the current state, and that the migration magnitudes derived from the overall partial connection number define the range of possible component values, the use of dynamic intervals—with defined midpoints and boundaries—is highly compatible with the mathematical structure of triangular fuzzy numbers [18]. Therefore, introducing triangular fuzzy numbers into CCD methods to characterize the dynamic value intervals both retains the most probable values and encompasses the uncertainty arising from microscopic migration between components. This approach also provides standardized input variables for subsequent stochastic simulations.

To address the limitations of existing coupling coordination degree evaluation methods in simultaneously ensuring the accuracy and reliability of evaluation results, this study proposes a novel evaluation method based on the logical multiplication of connection numbers and stochastic simulations (ECCD-LMS), building upon ECCD-SPR and ECCD-MCN. ECCD-LMS retains the basic idea underlying ECCD-SPR and ECCD-MCN, which use the sameness, difference, and opposition relationship information of connection numbers and their logical multiplication to characterize interactions among systems, and further extends deterministic coupling coordination degree evaluation to an evaluation process that incorporates uncertainty characterization and propagation. Specifically, ECCD-LMS introduces overall partial connection numbers based on the global structural constraint a+b+c=1 of three-element connection numbers to characterize the dynamic transformations among connection number components, and combines them with triangular fuzzy numbers to describe the dynamic value intervals of the comprehensive evaluation connection number components for each system. The Monte Carlo method is then employed for stochastic sampling, and the uncertainty in the connection number components of each system is propagated to the coupling coordination degree evaluation results of the composite system through the logical multiplication of connection numbers. In this way, ECCD-LMS extends the single deterministic coupling coordination degree values obtained by ECCD-SPR and ECCD-MCN to evaluation results comprising mean estimates and 95% uncertainty intervals, thereby simultaneously reflecting the coupling coordination level of the composite system and the uncertainty in the evaluation results. On this basis, the stability, evaluation performance, and uncertainty quantification capability of ECCD-LMS are comprehensively validated through convergence analysis of stochastic simulations, stochastic-simulation-based comparisons among different coupling coordination degree evaluation methods, sample-size stability analysis, and practical application to the WSE composite system in the Jing River Basin, with the aim of providing methodological support for reasonably assessing regional WSE coupling coordination levels under the combined influence of multiple sources of uncertainty.

## 2. Materials and Methods

### 2.1. Study Area

**Figure 1:**
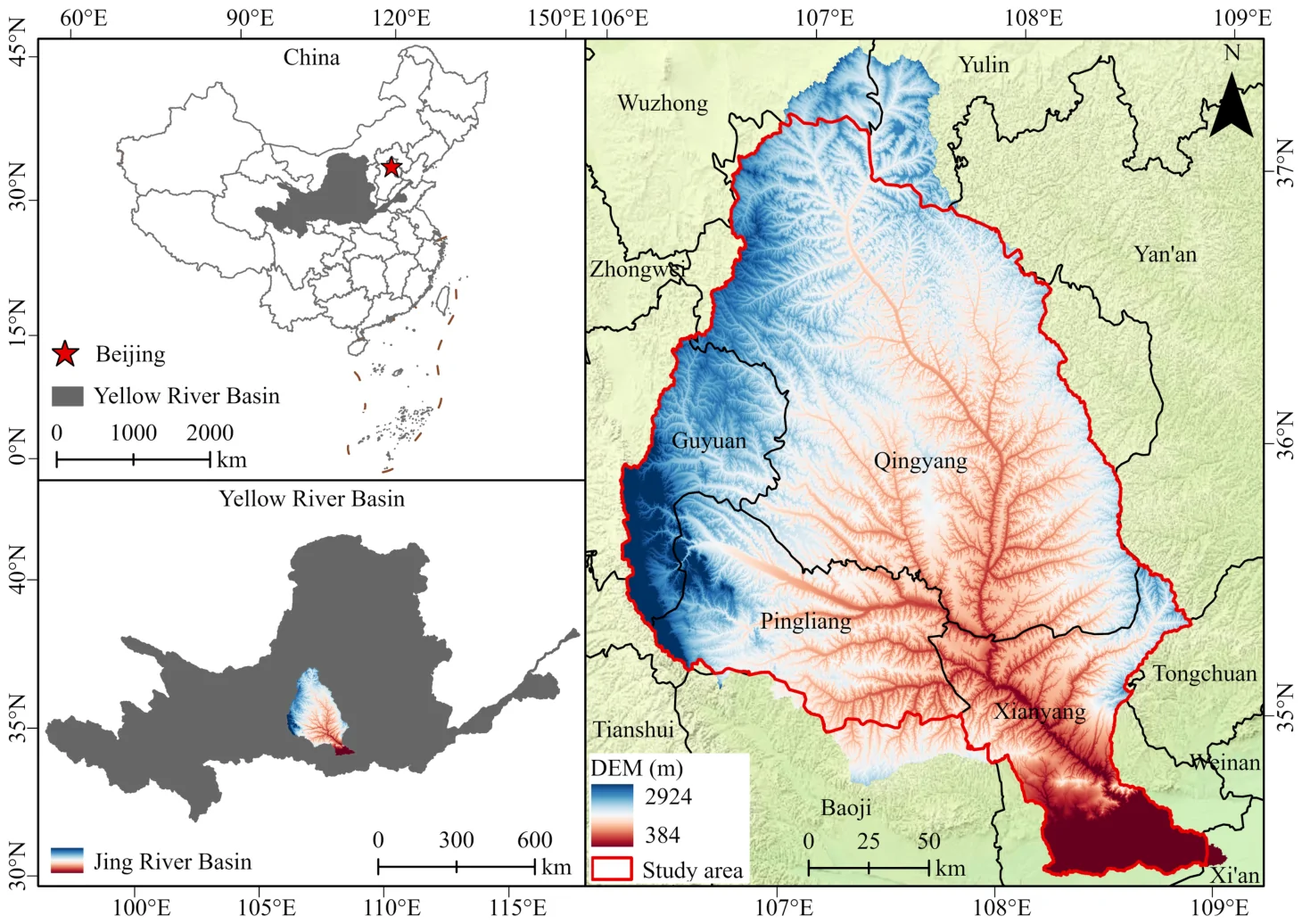
Location of the Jing River Basin and the four study subregions (Guyuan, Pingliang, Qingyang, and Xianyang) within the Yellow River Basin, China.

The Jing River, a primary tributary of the Wei River and a secondary tributary of the Yellow River, is located between 106°14′–108°42′ E and 34°46′–37°19′ N [19] (Figure 1). With a total length of 455 km, the Jing River originates from Laolongtan in the eastern foothills of the Liupan Mountains, flows from northwest to southeast through three province-level divisions (Ningxia, Gansu, and Shaanxi), and joins the Wei River in Gaoling District, Xi’an City [20]. The river basin covers an area of 45,421 km^2^, with an elevation ranging from 384 to 2924 m [21]. The basin is fan-shaped, featuring a well-developed river system and numerous tributaries, with major tributaries including the Malian River, Pu River, and Hei River.

The Jing River Basin spans 31 counties and districts under the jurisdiction of Guyuan City and Wuzhong City in the Ningxia Hui Autonomous Region; Pingliang City and Qingyang City in Gansu Province; and Baoji City, Xianyang City, Xi’an City, Tongchuan City, and Yulin City in Shaanxi Province [22]. Among these areas, the subregions of Guyuan, Pingliang, Qingyang, and Xianyang within the Jing River Basin account for approximately 39.59%, 64.66%, 88.03%, and 72.21% of the total area of their respective cities, and collectively cover approximately 94.13% of the total basin area. These subregions play a crucial role in the basin’s socioeconomic development and ecological environment protection. For evaluation purposes, the study area therefore focused on the Guyuan, Pingliang, Qingyang, and Xianyang subregions, as supported by data compiled from statistical yearbooks and water resources bulletins.

### 2.2. Data Sources

For this study, data on basic water resources, socioeconomic factors, and eco-environmental conditions were compiled for the four focal subregions in the Jing River Basin: Guyuan, Pingliang, Qingyang, and Xianyang. Water resources data were mainly derived from the Ningxia Water Resources Bulletin, Gansu Water Resources Bulletin, Shaanxi Water Resources Bulletin, and Xianyang Water Resources Bulletin from 2012 to 2023. Socioeconomic and eco-environmental data were primarily sourced from the 2012–2023 editions of the Ningxia Statistical Yearbook, Gansu Development Yearbook, Shaanxi Statistical Yearbook, Pingliang Development Yearbook, Qingyang Yearbook, Statistical Communiqué of Guyuan City on National Economic and Social Development, Statistical Communiqué of Pingliang City on National Economic and Social Development, Statistical Communiqué of Qingyang City on National Economic and Social Development, and Statistical Communiqué of Xianyang City on National Economic and Social Development.

### 2.3. Methods

This study sought to enhance the physical interpretability of CCD evaluation methods for the regional WSE composite system, improve the logical rationality of the evaluation process, and scientifically quantify uncertainty in the evaluation results. To achieve these goals, building upon ECCD-CN [14], ECCD-SPR [23], and ECCD-MCN [15], a novel CCD evaluation method was proposed based on the logical multiplication of connection numbers and stochastic simulations (ECCD-LMS). The ECCD-LMS method introduces the idea of mining uncertainty information by combining set pair analysis [24] with stochastic simulations [16]. In terms of the overall framework, ECCD-LMS consists of four stages: comprehensive evaluation of the individual systems, uncertainty characterization, uncertainty propagation, and determination of the evaluation results. First, set pair analysis [24] is used to calculate connection numbers for the water resources, social economy, and ecological environment systems. These connection numbers characterize each system based on the sample data, chosen evaluation indicators, grading criteria, and corresponding indicator weights. Next, overall partial connection numbers are calculated; these characterize the dynamics of individual connection number components (i.e., sameness, difference, and opposition degrees) for each system under the global constraint of *a* + *b* + *c* = 1. Based on the overall partial connection numbers, dynamic intervals are described for the triangular fuzzy number associated with each connection number component. The Monte Carlo method is then employed to stochastically sample within these intervals. Through the logical multiplication of connection numbers, the uncertainty associated with each individual system’s connection number components is propagated to the composite system’s CCD. Finally, stochastic simulations are used to determine means and uncertainty intervals for the evaluation results. As a result, the CCD is expanded from a single real value to a simulation-based uncertainty interval, thereby characterizing both the coupling coordination level and the uncertainty in the evaluation results. Building upon ECCD-SPR and ECCD-MCN, this framework explicitly characterizes the uncertainty inherent to the CCD evaluation process. Construction of the ECCD-LMS method includes the following six steps.

Step 1: Construct an evaluation index system and grading criteria.

An evaluation index system {$x_{ijk}$| *i* = 1, 2, …, $n_{i}$; *j* = 1, 2, …, $n_{j}$; *k* = 1, 2, …, $n_{jk}$} and grading criteria {$s_{qjk}$*| q* = 0, 1, …, $n_{g}$} are constructed to evaluate the CCD of the regional WSE composite system. This is based on established, general methods for constructing a comprehensive evaluation index system [25,26], relevant evaluation criteria, and existing research [27], as well as the coupling, synergistic, and mutual-feedback relationships among regional water resources, socioeconomic development, and eco-environmental conditions [2,28] and the functional characteristics of the evaluation indicators within the composite system. Here, $x_{ijk}$ represents the value of an evaluation indicator, and $s_{qjk}$ denotes the value of a grading criterion for indicator *k* in system *j*. The subscripts *i*, *j*, and *k* are indices associated with the evaluation sample, the system, and the indicator, respectively; *g* indicates the *g*-th evaluation grade for indicator *k* in system *j*. The total number of evaluation samples, systems, and indicators is represented by $n_{i}$, $n_{j}$, and $n_{jk}$, respectively, while $n_{g}$ is the total number of evaluation grades.

In this study, each indicator was divided into three grades (i.e., $n_{g}$ = 3). Grade I represents a coordinated state, indicating that the current status of each indicator fully satisfies requirements for the coordinated development of the corresponding systems, and that the composite system is in a coordinated development stage. Grade II represents a marginally coordinated state, wherein interactions among systems are approaching a coordinated development stage. Grade III represents an uncoordinated state, where the current status of individual indicators in one system constrains the sustainable development of corresponding systems; thus, the composite system is in an uncoordinated development stage.

Step 2: Calculate single-indicator connection numbers ($u_{ijk}$) for the evaluation sample.

Set pair analysis (SPA), a method proposed by the Chinese scholar Keqin Zhao in 1989 [24], integrates the certainty and uncertainty of a problem into a unified conclusion. This method can be used to investigate the internal relationships between a system and its constituent elements. The connection number is a mathematical tool used to quantify these relationships and is expressed as follows [29,30]:

(1) u=a+bI+cJ,

where *a*, *b*, and *c* represent the sameness, difference, and opposition degrees, respectively, with the values of each ranging from zero to one; and *I* and *J* are the difference coefficient and opposition coefficient, respectively. Since the connection numbers in this study belong to the positive-negative type, *I* ∈ [−1, 1] and *J* = −1 [24].

Set pair analysis [23,24] is applied to calculate the connection number $u_{ijkg}$ between the evaluation indicator value $x_{ijk}$ and the grading criterion values $s_{qjk}$. For positive indicators that increase with the evaluation grade (*g*), the connection numbers for each grade are calculated as follows:

(2) $$u_{ijk1}=\left\{\begin{array}{cc} 1, & x_{ijk}\in \left[s_{0jk},s_{1jk}\right],\\ 1-\frac{2(x_{ijk}-s_{1jk})}{s_{2jk}-s_{1jk}}, & x_{ijk}\in \left(s_{1jk},s_{2jk}\right],\\ -1, & x_{ijk}\in \left(s_{2jk},s_{3jk}\right]; \end{array}\right.$$

(3) $$u_{ijk2}=\left\{\begin{array}{cc} 1-\frac{2(s_{1jk}-x_{ijk})}{s_{1jk}-s_{0jk}}, & x_{ijk}\in \left[s_{0jk},s_{1jk}\right],\\ 1, & x_{ijk}\in \left(s_{1jk},s_{2jk}\right],\\ 1-\frac{2(x_{ijk}-s_{2jk})}{s_{3jk}-s_{2jk}}, & x_{ijk}\in \left(s_{2jk},s_{3jk}\right],\\ -1, & x_{ijk}\in \left(s_{3jk},+\infty \right); \end{array}\right.$$

(4) $$u_{ijk3}=\left\{\begin{array}{cc} -1, & x_{ijk}\in \left[s_{0jk},s_{1jk}\right],\\ 1-\frac{2(s_{2jk}-x_{ijk})}{s_{2jk}-s_{1jk}}, & x_{ijk}\in \left(s_{1jk},s_{2jk}\right],\\ 1, & x_{ijk}\in \left(s_{2jk},s_{3jk}\right], \end{array}\right.$$

where $s_{0jk}$ to $s_{3jk}$ represent boundary values defining the evaluation grades (Grade 1 to Grade 3) [23]. For negative indicators that decrease as the evaluation grade increases, $x_{ijk}$ and $s_{qjk}$ are negated before being substituted into the above equations (Equations (2)–(4)).

Since the positive-negative connection number component $u_{ijkg}$ ranges from −1 to 1 [24], a relative membership degree formula is applied for conversion to a positive scale. After conversion, the relative membership degree of the evaluation indicator value $x_{ijk}$ belonging to the fuzzy set for evaluation grade *g* ($v_{ijkg}^{*}$) falls within the interval from zero to one [13,31]:

(5) $$v_{ijkg}^{*}=\frac{1}{2}+\frac{1}{2}u_{ijkg}.$$

By normalizing Equation (5), the components $v_{ijk1}$, $v_{ijk2}$, and $v_{ijk3}$ of the single-indicator connection number can be obtained for CCD evaluation of the WSE composite system [24]:

(6) $$v_{ijkg}=\frac{v_{ijkg}^{*}}{\sum_{g=1}^{3}v_{ijkg}^{*}}.$$

Finally, the single-indicator connection number $u_{ijk}$ is constructed as follows [24]:

(7) $$u_{ijk}=v_{ijk1}+v_{ijk2}I+v_{ijk3}J=a+bI+cJ.$$

Step 3: Calculate the comprehensive connection numbers $u_{i1}$, $u_{i2}$, and $u_{i3}$ for systems.

The comprehensive evaluation of a system is a holistic assessment of the degree to which its evaluation objects conform to the composite system’s overall goal (i.e., coupling coordinated development) [8]. Given the impact of sampling uncertainty associated with evaluation indicator values, as well as uncertainty in comparing indicator values to grading criteria values during the evaluation process, the comprehensive evaluation of each system also possesses some level of uncertainty. Drawing upon concepts from set pair reasoning [8], the water resources, social economy, and ecological environment systems can be comprehensively evaluated using three-element connection numbers. By applying a weight to each indicator ($w_{k}$), the single-indicator connection numbers $u_{ijk}$ from Equation (7) can be transformed into the comprehensive evaluation connection numbers $u_{i1}$, $u_{i2}$, and $u_{i3}$ (for water resources, social economy, and ecological environment systems, respectively). This is achieved via a weighted summation approach [32]:

(8) $$u_{i1}=a_{i1}+b_{i1}I+c_{i1}J=\sum_{k=1}^{n_{1k}}w_{k}v_{i1k1}+\sum_{k=1}^{n_{1k}}w_{k}v_{i1k2}I+\sum_{k=1}^{n_{1k}}w_{k}v_{i1k3}J;$$

(9) $$u_{i2}=a_{i2}+b_{i2}I+c_{i2}J=\sum_{k=1}^{n_{2k}}w_{k}v_{i2k1}+\sum_{k=1}^{n_{2k}}w_{k}v_{i2k2}I+\sum_{k=1}^{n_{2k}}w_{k}v_{i2k3}J;$$

(10) $$u_{i3}=a_{i3}+b_{i3}I+c_{i3}J=\sum_{k=1}^{n_{3k}}w_{k}v_{i3k1}+\sum_{k=1}^{n_{3k}}w_{k}v_{i3k2}I+\sum_{k=1}^{n_{3k}}w_{k}v_{i3k3}J,$$

where $a_{i1}$ + $b_{i1}$*I* + $c_{i1}$*J*, $a_{i2}$ + $b_{i2}$*I* + $c_{i2}$*J*, and $a_{i3}$ + $b_{i3}$*I* + $c_{i3}$*J* represent the three-element connection numbers for the comprehensive evaluation of the three respective systems; and $n_{1k}$, $n_{2k}$, and $n_{3k}$ denote the number of evaluation indicators within each system, satisfying the condition $n_{1k}$ + $n_{2k}$ + $n_{3k}$ = $n_{k}$, where $n_{k}$ is the total number of CCD evaluation indicators.

Step 4: Construct dynamic value intervals for each system’s comprehensive evaluation connection number components based on overall partial connection numbers and triangular fuzzy numbers.

From a macroscopic perspective, the comprehensive evaluation connection number components for each system, $a_{ij}$, $b_{ij}$, and $c_{ij}$, reflect the extent of same, different, and opposite relationships within the water resources, social economy, and ecological environment systems, respectively. In the context of CCD evaluation, these components ($a_{ij}$, $b_{ij}$, and $c_{ij}$) represent the degree of conformity between evaluation indicator values and evaluation grades within a given system. Currently, most studies directly substitute these components into the equation for set pair potential to construct evaluation methods. However, according to SPA theory, including principles such as the universality of contradiction and the absoluteness of motion, the connection number components are not independent of one another. Instead, they undergo certain microscopic migrations and mutual transformations during the evolution of the system state [16]. Partial connection numbers [17] and semi-partial connection numbers [16] focus on the dynamics of the relational structure of set pair systems, and they can quantify micro-level movements among connection number components. For a three-element connection number, the corresponding first-order positive and negative semi-partial connection numbers, denoted by $\partial_{\mathrm{sp}}^{+}\mu$ and $\partial_{\mathrm{sp}}^{-}\mu$, respectively, are calculated as follows [16]:

(11) $$\partial_{\mathrm{sp}}^{+}\mu =\partial_{\mathrm{sp}}^{+}a+\partial_{\mathrm{sp}}^{+}bi_{\mathrm{sp}}^{+}=\frac{a}{a+b}+\frac{b}{b+c}i_{\mathrm{sp}}^{+};$$

(12) $$\partial_{\mathrm{sp}}^{-}\mu =\partial_{\mathrm{sp}}^{-}bi_{\mathrm{sp}}^{-}+\partial_{\mathrm{sp}}^{-}cj_{\mathrm{sp}}=\frac{b}{a+b}i_{\mathrm{sp}}^{-}+\frac{c}{b+c}j_{\mathrm{sp}},$$

where $\partial_{\mathrm{sp}}^{+}a=\frac{a}{a+b}$, $\partial_{\mathrm{sp}}^{+}b=\frac{b}{b+c}$, $\partial_{\mathrm{sp}}^{-}b=\frac{b}{a+b}$, and $\partial_{\mathrm{sp}}^{-}c=\frac{c}{b+c}$ are all semi-partial connection numbers. Here, $\partial_{\mathrm{sp}}^{+}a$ and $\partial_{\mathrm{sp}}^{+}b$ represent the positive migration rates. In other words, they quantify transformations from *b* to *a* and from *c* to *b*, respectively. Conversely, $\partial_{\mathrm{sp}}^{-}b$ and $\partial_{\mathrm{sp}}^{-}c$ represent the negative migration rates, capturing transformations from *a* to *b* and from *b* to *c*, respectively. $i_{\mathrm{sp}}^{+}$ and $i_{\mathrm{sp}}^{-}$ are uncertainty coefficients, each taking values in the interval [−1,1]; $j_{\mathrm{sp}}$ is the indicative coefficient and is generally taken as −1.

However, the semi-partial connection numbers in Equations (11) and (12) are normalized only within the adjacent component pairs a+b and b+c, and thus do not explicitly account for the global structural constraint a+b+c=1 when multiple migration directions occur simultaneously. Because *a*, *b*, and *c* are normalized components of the same connection number and satisfy a+b+c=1, their mutual transformations represent redistribution within a fixed overall relational structure rather than an increase in the total amount of the three components. For example, when *b* simultaneously migrates toward *a* and *c*, the corresponding migration rates are $\frac{a}{a+b}$ and $\frac{c}{b+c}$, respectively. Their sum satisfies $\frac{a}{a+b}+\frac{c}{b+c}-1=\frac{ac-b^{2}}{\left(a+b)(b+c\right)}$. Therefore, when $ac>b^{2}$, the sum of the two migration rates exceeds 1, meaning that the total amount assigned to the two outward migrations of *b* can exceed *b* itself. This indicates that local pairwise normalization may overestimate the magnitude of simultaneous transformations among the connection number components and may consequently cause the transformed connection number to deviate from the interval [−1,1] [16]. To ensure that the simultaneous transformations remain consistent with the global constraint of the connection number structure, overall partial connection numbers were proposed by using a+b+c as the common denominator [16]. Under the constraint a+b+c=1, the combined migration proportion from *b* toward *a* and *c* becomes $\frac{a}{a+b+c}+\frac{c}{a+b+c}=a+c=1-b\leq 1$, so that the total migration amount of *b* remains bounded by its available component value. Accordingly, the overall partial connection numbers, denoted by $\partial_{\mathrm{op}}^{+}\mu$ and $\partial_{\mathrm{op}}^{-}\mu$, are calculated as follows:

(13) $$\partial_{\mathrm{op}}^{+}\mu =\partial_{\mathrm{op}}^{+}a+\partial_{\mathrm{op}}^{+}bi_{\mathrm{op}}^{+}=\frac{a}{a+b+c}+\frac{b}{a+b+c}i_{\mathrm{op}}^{+};$$

(14) $$\partial_{\mathrm{op}}^{-}\mu =\partial_{\mathrm{op}}^{-}bi_{\mathrm{op}}^{-}+\partial_{\mathrm{op}}^{-}cj_{\mathrm{op}}=\frac{b}{a+b+c}i_{\mathrm{op}}^{-}+\frac{c}{a+b+c}j_{\mathrm{op}},$$

where $\partial_{\mathrm{op}}^{+}a=\frac{a}{a+b+c}$ and $\partial_{\mathrm{op}}^{+}b=\frac{b}{a+b+c}$ are the overall partial positive connection numbers, as calculated based on the sum of the sameness, difference, and opposition components (*a* + *b* + *c*); and $\partial_{\mathrm{op}}^{-}b=\frac{b}{a+b+c}$ and $\partial_{\mathrm{op}}^{-}c=\frac{c}{a+b+c}$ are the overall partial negative connection numbers, also calculated based on the sum *a* + *b* + *c*. $i_{\mathrm{op}}^{+}$ and $i_{\mathrm{op}}^{-}$ are uncertainty coefficients, each taking values in the interval [−1,1]; $j_{\mathrm{op}}$ is the indicative coefficient and is generally taken as −1.

Based on the overall partial connection numbers, the dynamic value intervals of the connection number components are constructed by considering their mutual migration paths. According to the transformation relationships represented by the overall partial connection numbers, migrations occur between adjacent components following a↔b↔c, whereas transformations between *a* and *c* proceed successively through *b*. Taking *a* as an example, migration toward *a* consists of the direct migration b→a and the successive migration c→b→a, whereas migration away from *a* consists of a→b and a→b→c. Accordingly, the corresponding migration magnitudes are $b\frac{a}{a+b+c}+c\frac{b}{a+b+c}\frac{a}{a+b+c}$ and $a\frac{b}{a+b+c}+a\frac{b}{a+b+c}\frac{c}{a+b+c}$, respectively. The migration magnitudes toward and away from *b* and *c* can be obtained analogously. The original values of *a*, *b*, and *c* are regarded as the most probable values under the current system state, while the migration magnitudes away from and toward each component determine its lower and upper bounds, respectively. Therefore, by incorporating triangular fuzzy number theory [18], the dynamic value intervals of $a^{\prime }$, $b^{\prime }$, and $c^{\prime }$ can be expressed as follows [16]:

Sameness degree $a^{\prime }$:
  (15) $$\left(a-a\frac{b}{a+b+c}-a\frac{b}{a+b+c}\cdot \frac{c}{a+b+c},\;a,\;a+b\frac{a}{a+b+c}+c\frac{b}{a+b+c}\cdot \frac{a}{a+b+c}\right);$$
  Difference degree $b^{\prime }$:
  (16) $$\left(b-b\frac{a}{a+b+c}-b\frac{c}{a+b+c},\;b,\;b+a\frac{b}{a+b+c}+c\frac{b}{a+b+c}\right);$$
  Opposition degree $c^{\prime }$:
  (17) $$\left(c-c\frac{b}{a+b+c}-c\frac{b}{a+b+c}\cdot \frac{a}{a+b+c},\;c,\;c+b\frac{c}{a+b+c}+a\frac{c}{a+b+c}\cdot \frac{b}{a+b+c}\right).$$

Since *a* + *b* + *c* = 1 in the three-element connection number, Equations (15)–(17) can be simplified to:

(18) $$\mathrm{Sameness}\;\mathrm{degree}\;a^{\prime }:\;\left(a-ab-abc,\;a,\;a+ba+cba\right);$$

(19) $$\mathrm{Difference}\;\mathrm{degree}\;b^{\prime }:\;\left(b-ba-bc,\;b,\;b+ab+cb\right);$$

(20) $$\mathrm{Opposition}\;\mathrm{degree}\;c^{\prime }:\;\left(c-cb-cba,\;c,\;c+bc+abc\right).$$

Letting Δa=ab+abc, Δb=ab+bc, and Δc=cb+cba, Equations (18)–(20) can be further simplified as follows: sameness degree (a−Δa, *a*, a+Δa), difference degree (b−Δb, *b*, b+Δb), and opposition degree (c−Δc, *c*, c+Δc). By substituting the above simplified formulae into those for the comprehensive evaluation connection numbers for each system from Step 3, the triangular fuzzy numbers for the sameness, difference, and opposition degrees of sample *i* in system *j* can be obtained as $T_{ij}^{\left(a\right)}=\left(a_{ij}^{L},a_{ij},a_{ij}^{R}\right)$, $T_{ij}^{\left(b\right)}=\left(b_{ij}^{L},b_{ij},b_{ij}^{R}\right)$, and $T_{ij}^{\left(c\right)}=\left(c_{ij}^{L},c_{ij},c_{ij}^{R}\right)$, respectively, where $a_{ij}^{L}=a_{ij}-\Delta a_{ij}$, $a_{ij}^{R}=a_{ij}+\Delta a_{ij}$, $b_{ij}^{L}=b_{ij}-\Delta b_{ij}$, $b_{ij}^{R}=b_{ij}+\Delta b_{ij}$, $c_{ij}^{L}=c_{ij}-\Delta c_{ij}$, and $c_{ij}^{R}=c_{ij}+\Delta c_{ij}$. Since the connection number components satisfy $a_{ij},b_{ij},c_{ij}\in \left[0,1\right]$ [24] and $a_{ij}+b_{ij}+c_{ij}=1$, the resulting interval endpoints all lie within [0,1].

Step 5: Using stochastic simulations, determine uncertainty intervals for the estimated CCD.

To investigate how uncertainty in the comprehensive evaluation of each system, as quantified in Step 4 (by triangular fuzzy numbers), propagates through the logical multiplication of connection numbers and ultimately affects estimates of CCD ($D_{i}$) in the composite system, the Monte Carlo algorithm [33] is used to simulate this process. For system *j*, the triangular fuzzy numbers representing the sameness, difference, and opposition components of the comprehensive evaluation connection number are $T_{ij}^{\left(a\right)}$, $T_{ij}^{\left(b\right)}$, and $T_{ij}^{\left(c\right)}$, respectively, as defined in Step 4, where $a_{ij}^{L}\leq a_{ij}\leq a_{ij}^{R}$, $b_{ij}^{L}\leq b_{ij}\leq b_{ij}^{R}$, and $c_{ij}^{L}\leq c_{ij}\leq c_{ij}^{R}$. Let *M* be the total number of simulations. In the *m*-th simulation (*m* = 1, 2, …, *M*), three mutually independent random numbers, each uniformly distributed over [0, 1], are generated for the water resources, social economy, and ecological environment systems, respectively, and are denoted by $\xi_{i1m}$, $\xi_{i2m}$, and $\xi_{i3m}$. Next, using the dynamic value intervals for the three systems—water resources, social economy, and ecological environment—determined via Equations (18)–(20) in Step 4, random sampling is performed to obtain a set of pre-normalization samples of the connection number components (${\tilde{a}}_{ijm}$, ${\tilde{b}}_{ijm}$, and ${\tilde{c}}_{ijm}$) for system *j* [16]:

(21) $${\tilde{a}}_{ijm}=\left\{\begin{array}{cc} a_{ij}^{L}+\sqrt{\xi_{ijm}\left(a_{ij}-a_{ij}^{L}\right)\left(a_{ij}^{R}-a_{ij}^{L}\right)},\; & \xi_{ijm}\leq \frac{a_{ij}-a_{ij}^{L}}{a_{ij}^{R}-a_{ij}^{L}},\\ a_{ij}^{R}-\sqrt{\left(1-\xi_{ijm}\right)\left(a_{ij}^{R}-a_{ij}\right)\left(a_{ij}^{R}-a_{ij}^{L}\right)},\; & \xi_{ijm}>\frac{a_{ij}-a_{ij}^{L}}{a_{ij}^{R}-a_{ij}^{L}}. \end{array}\right.$$

(22) $${\tilde{b}}_{ijm}=\left\{\begin{array}{cc} b_{ij}^{L}+\sqrt{\xi_{ijm}\left(b_{ij}-b_{ij}^{L}\right)\left(b_{ij}^{R}-b_{ij}^{L}\right)},\; & \xi_{ijm}\leq \frac{b_{ij}-b_{ij}^{L}}{b_{ij}^{R}-b_{ij}^{L}},\\ b_{ij}^{R}-\sqrt{\left(1-\xi_{ijm}\right)\left(b_{ij}^{R}-b_{ij}\right)\left(b_{ij}^{R}-b_{ij}^{L}\right)},\; & \xi_{ijm}>\frac{b_{ij}-b_{ij}^{L}}{b_{ij}^{R}-b_{ij}^{L}}. \end{array}\right.$$

(23) $${\tilde{c}}_{ijm}=\left\{\begin{array}{cc} c_{ij}^{L}+\sqrt{\xi_{ijm}\left(c_{ij}-c_{ij}^{L}\right)\left(c_{ij}^{R}-c_{ij}^{L}\right)},\; & \xi_{ijm}\leq \frac{c_{ij}-c_{ij}^{L}}{c_{ij}^{R}-c_{ij}^{L}},\\ c_{ij}^{R}-\sqrt{\left(1-\xi_{ijm}\right)\left(c_{ij}^{R}-c_{ij}\right)\left(c_{ij}^{R}-c_{ij}^{L}\right)},\; & \xi_{ijm}>\frac{c_{ij}-c_{ij}^{L}}{c_{ij}^{R}-c_{ij}^{L}}. \end{array}\right.$$

Equations (21)–(23) implement inverse transform sampling from triangular distributions. Taking Equation (21) as an example, a uniformly distributed random number $\xi_{ijm}$ over [0, 1] is transformed into a sample from the triangular fuzzy number $\left(a_{ij}^{L},a_{ij},a_{ij}^{R}\right)$ through the inverse of its cumulative distribution function (CDF). The threshold $\left(a_{ij}-a_{ij}^{L}\right)/\left(a_{ij}^{R}-a_{ij}^{L}\right)$ is exactly the CDF value evaluated at the mode $a_{ij}$: when $\xi_{ijm}$ does not exceed this threshold, the sampled value falls on the left (rising) branch of the triangle, between $a_{ij}^{L}$ and $a_{ij}$; otherwise, it falls on the right (falling) branch, between $a_{ij}$ and $a_{ij}^{R}$. Consequently, the pre-normalization samples ${\tilde{a}}_{ijm}$, ${\tilde{b}}_{ijm}$, and ${\tilde{c}}_{ijm}$ concentrate around the most likely values ($a_{ij}$, $b_{ij}$, $c_{ij}$), with the sampling probability decreasing linearly toward the interval endpoints. These pre-normalization samples are subsequently normalized within each system using a normalization procedure analogous to that in Equation (6). The normalized components, denoted by $a_{ijm}^{\prime }$, $b_{ijm}^{\prime }$, and $c_{ijm}^{\prime }$, satisfy $a_{ijm}^{\prime }+b_{ijm}^{\prime }+c_{ijm}^{\prime }=1$.

After normalization, comprehensive evaluation connection numbers are calculated for each system for the *m*-th simulation: $u_{i1m}=a_{i1m}^{\prime }+b_{i1m}^{\prime }I+c_{i1m}^{\prime }J$, $u_{i2m}=a_{i2m}^{\prime }+b_{i2m}^{\prime }I+c_{i2m}^{\prime }J$, and $u_{i3m}=a_{i3m}^{\prime }+b_{i3m}^{\prime }I+c_{i3m}^{\prime }J$. The normalized connection number components are also substituted into Equation (24) for combination via logical multiplication. To satisfy the normalization condition, further processing is performed through Equations (25) and (26) to obtain a normalized three-element connection number ($u_{im}$) that characterizes the coupling coordination level of the regional WSE composite system for the *m*-th simulation [8]:

(24) $$\begin{array}{cc} U_{im}^{*}= & \sqrt[{3}]{a_{i1m}^{\prime }a_{i2m}^{\prime }a_{i3m}^{\prime }}+\frac{1}{2}\left[\sqrt[{3}]{b_{i1m}^{\prime }b_{i2m}^{\prime }b_{i3m}^{\prime }}+\frac{1}{3}\left(b_{i1m}^{\prime }+b_{i2m}^{\prime }+b_{i3m}^{\prime }\right)\right]I\\ \; & +\frac{1}{3}\left(c_{i1m}^{\prime }+c_{i2m}^{\prime }+c_{i3m}^{\prime }\right)J; \end{array}$$

(25) $$\begin{array}{cc} Z_{im}= & \sqrt[{3}]{a_{i1m}^{\prime }a_{i2m}^{\prime }a_{i3m}^{\prime }}+\frac{1}{2}\left[\sqrt[{3}]{b_{i1m}^{\prime }b_{i2m}^{\prime }b_{i3m}^{\prime }}+\frac{1}{3}\left(b_{i1m}^{\prime }+b_{i2m}^{\prime }+b_{i3m}^{\prime }\right)\right]\\ \; & +\frac{1}{3}\left(c_{i1m}^{\prime }+c_{i2m}^{\prime }+c_{i3m}^{\prime }\right); \end{array}$$

(26) $$\begin{array}{cc} U_{im}= & \frac{1}{Z_{im}}\sqrt[{3}]{a_{i1m}^{\prime }a_{i2m}^{\prime }a_{i3m}^{\prime }}+\frac{1}{2Z_{im}}\left[\sqrt[{3}]{b_{i1m}^{\prime }b_{i2m}^{\prime }b_{i3m}^{\prime }}+\frac{1}{3}\left(b_{i1m}^{\prime }+b_{i2m}^{\prime }+b_{i3m}^{\prime }\right)\right]I\\ \; & +\frac{1}{3Z_{im}}\left(c_{i1m}^{\prime }+c_{i2m}^{\prime }+c_{i3m}^{\prime }\right)J. \end{array}$$

Since the subtraction set pair potential of the three-element connection number in Equation (26) [23] has a range of [−1, 1], a linear transformation is applied to map the set pair potential to the conventional interval [0, 1] for CCD evaluation results, yielding the CCD ($D_{im}$) of the composite system for the current simulation:

(27) $$\begin{array}{cc} D_{im}= & \frac{1}{2}+\frac{1}{2}\left[\frac{1}{Z_{im}}\sqrt[{3}]{a_{i1m}^{\prime }a_{i2m}^{\prime }a_{i3m}^{\prime }}-\frac{1}{3Z_{im}}\left(c_{i1m}^{\prime }+c_{i2m}^{\prime }+c_{i3m}^{\prime }\right)\right]\\ \; & \times \left\{1+\frac{1}{2Z_{im}}\left[\sqrt[{3}]{b_{i1m}^{\prime }b_{i2m}^{\prime }b_{i3m}^{\prime }}+\frac{1}{3}\left(b_{i1m}^{\prime }+b_{i2m}^{\prime }+b_{i3m}^{\prime }\right)\right]\right\}. \end{array}$$

Through Equations (24)–(27), a total of *M* CCD values ($D_{im}$) are obtained. All *M* simulation results are sorted in ascending order. If the value at the *l*-th position is denoted as $D_{i(l)}$, the corresponding empirical cumulative probability ($P_{l}$) can be defined as:

(28) $$P_{l}=\frac{l}{M+1}.$$

Since a higher rank (*l*) after sorting corresponds to a larger CCD value, the lower and upper bounds of a central 100(1−α)% simulation-based uncertainty interval can be determined directly from the empirical distribution [34], where α denotes the total probability assigned to the two tails (α = 0.05 in this study). Specifically, the lower uncertainty bound $D_{i}^{L}$ and upper uncertainty bound $D_{i}^{U}$ correspond to the empirical quantiles at probabilities 0.5α and 1−0.5α, respectively. Based on Equation (28), their corresponding ranks are *INT*(0.5α(*M* + 1)) and *INT*((1−0.5α)(*M* + 1)), respectively. That is:

(29) $$\left[D_{i}^{L},D_{i}^{U}\right]=\left[D_{i[\mathrm{INT}(0.5\alpha (M+1))]},D_{i[\mathrm{INT}((1-0.5\alpha )(M+1))]}\right],$$

where the interval $\left[D_{i}^{L},D_{i}^{U}\right]$ characterizes the range of CCD estimates under the combined effect of multiple sources of uncertainty.

Step 6: Determine evaluation grades for CCD.

Following commonly used grading criteria for CCD [35,36,37] and using the principle of equal division, in this study, the CCD value interval [0, 1] was divided into three development stages comprising a total of 10 evaluation grades, as shown in Table 1. After obtaining the uncertainty interval for CCD, its lower and upper bounds are categorized according to the grading criteria shown in Table 1. If both bounds correspond to the same evaluation grade, the CCD is considered to remain within that grade across the uncertainty interval. If the bounds correspond to different grades, the uncertainty interval spans one or more grade boundaries. To intuitively reflect this uncertainty, this study adopted a “combined grade” approach in these cases, denoting the evaluation result as a combination of the grades corresponding to the two bounds. For example, when $D_{i}^{L}$ corresponds to “extreme incoordination” and $D_{i}^{U}$ to “severe incoordination”, the state is denoted as “extreme–severe incoordination”, thereby concisely indicating the range of evaluation grades covered by the 95% uncertainty interval.

## 3. Results

### 3.1. Evaluation Index System and Grading Criteria

Based on the interactions among the water resources, social economy, and ecological environment systems in the Jing River Basin, and with reference to approaches used in previous studies to construct evaluation index systems [38,39,40], an evaluation index system was constructed to evaluate the CCD of the WSE composite system. Four indicators were selected for the water resources system, eight for the social economy system, and eight for the ecological environment system. The grading thresholds for each indicator were determined by taking into account the indicator attributes, the actual conditions of the Jing River Basin, and the opinions of experts in the relevant research fields. The indicator weights were adopted from Ref. [27], as shown in Table 2.

### 3.2. Distribution of Comprehensive Evaluation Connection Numbers for Water Resources, Social Economy, and Ecological Environment Systems in the Jing River Basin

**Figure 2:**
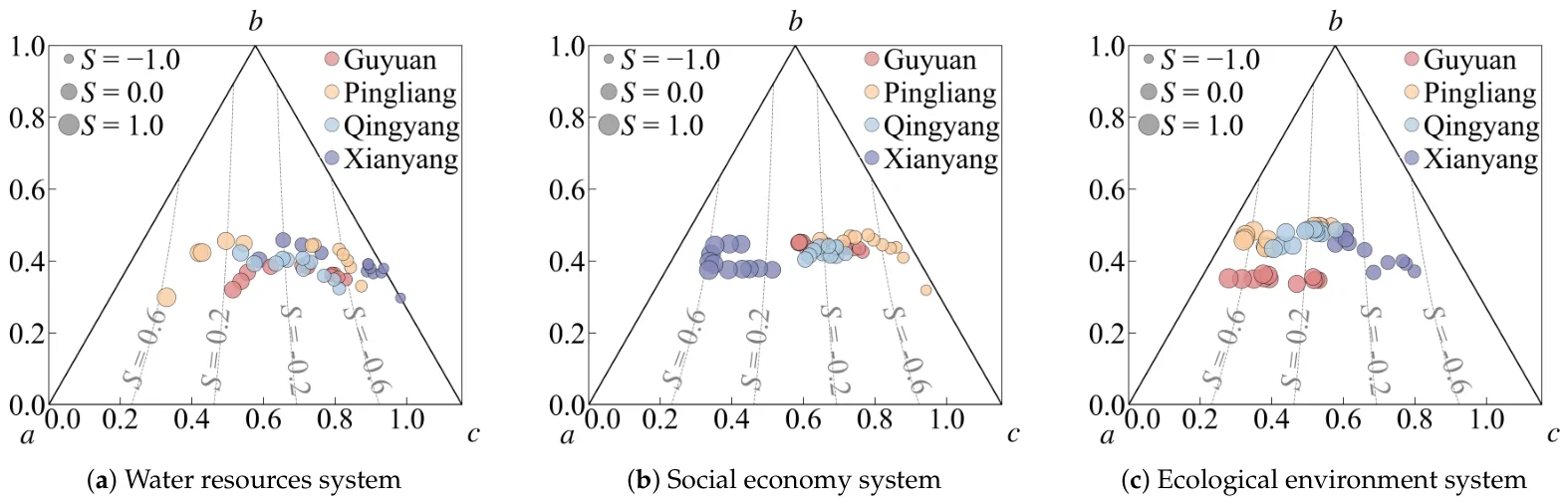
Distribution of comprehensive evaluation connection numbers for the water resources, social economy, and ecological environment systems in the four subregions of the Jing River Basin from 2012 to 2023. Each point corresponds to a comprehensive evaluation connection number. The perpendicular distances from a given point to side *bc*, *ac*, and *ab* of the triangle correspond to sameness (*a*), difference (*b*), and opposition (*c*) degrees, respectively. The subtraction set pair potential of the connection numbers is denoted by *S*. The size of each point represents the value of the corresponding subtraction set pair potential. The dashed lines divide the triangle into five regions, which represent (from left to right): same potential (0.6 < *S* ≤ 1), partial same potential (0.2 < *S* ≤ 0.6), balance potential (−0.2 ≤ *S* ≤ 0.2), partial anti-potential (−0.6 ≤ *S* < −0.2), and anti-potential (−1 ≤ *S* < −0.6) [41].

Data for the 20 evaluation indicators listed in Table 2 were obtained for the four focal subregions—Guyuan, Pingliang, Qingyang, and Xianyang—for the years 2012 to 2023 from statistical yearbooks, water resources bulletins, and other sources. These values were substituted into Equations (2)–(7) to obtain the single-indicator connection numbers ($u_{ijk}$), and then into Equations (8)–(10) to obtain the comprehensive evaluation connection numbers for the water resources ($u_{i1}$), social economy ($u_{i2}$), and ecological environment ($u_{i3}$) systems for the corresponding years, as shown in Figure 2.

As can be seen from Figure 2, due to variations in the development of water resources, social economy, and ecological environment systems among Guyuan, Pingliang, Qingyang, and Xianyang, the distribution of comprehensive evaluation connection numbers differed considerably among the subregions. The water resources system (Figure 2a) was affected by factors such as spatiotemporal heterogeneity in precipitation, resulting in a relatively wide range of connection numbers for all four subregions. In terms of the overall distribution, the comprehensive evaluation connection numbers spanned multiple potential grades from “partial same potential” to “anti-potential”, indicating that the development status of water resources within the Jing River Basin was highly unstable. Notably, the connection numbers for Xianyang are concentrated on the right side of the triangle in Figure 2a, with disproportionately high opposition degree values. This suggests that the development of water resources was limited across Xianyang, representing a critical constraint on the coordinated development of the composite system. As compared to the water resources system, the comprehensive evaluation connection numbers were relatively concentrated for each subregion for the social economy (Figure 2b) and ecological environment (Figure 2c) systems; in areas with higher evaluation levels for the social economy system, the ecological environment tended to face greater pressure, and vice versa. For the social economy system, the comprehensive evaluation connection numbers for Xianyang are concentrated on the left side of the triangle in Figure 2b, indicating a higher development level across Xianyang; whereas those for Guyuan, Pingliang, and Qingyang are clustered on the right side of the triangle, suggesting a relative lag in development. However, in the ecological environment system, this pattern is reversed: Guyuan, Pingliang, and Qingyang are now clustered on the left side of the triangle (Figure 2c), while samples from economically well-developed Xianyang are clustered on the right side.

### 3.3. Spatiotemporal Variability in CCD for the WSE Composite System of the Jing River Basin

**Figure 3:**
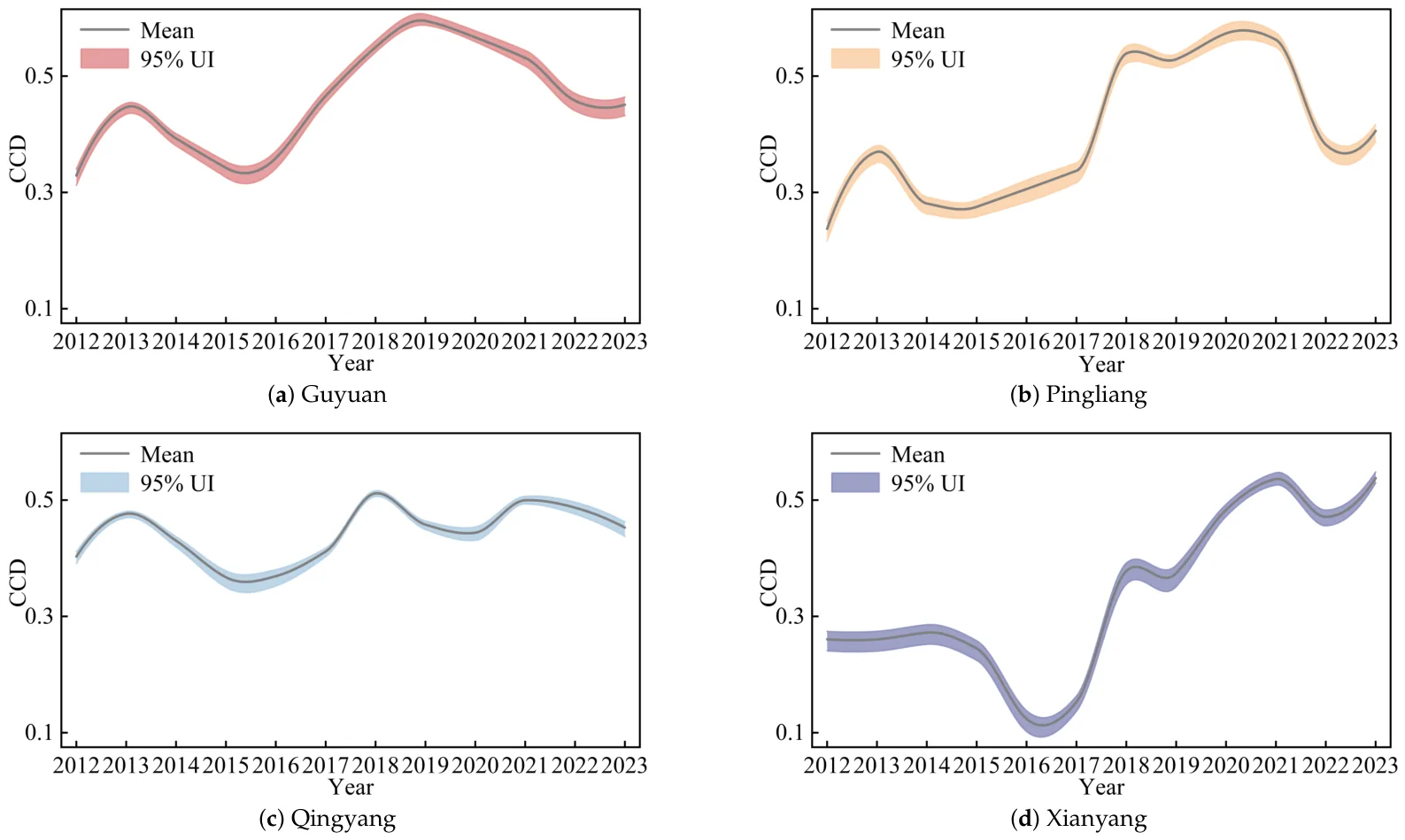
Mean coupling coordination degree (CCD) with 95% uncertainty interval (UI) for the water resources, social economy, and ecological environment (WSE) composite system in the four subregions of the Jing River Basin from 2012 to 2023.

The comprehensive evaluation connection numbers for the water resources ($u_{i1}$), social economy ($u_{i2}$), and ecological environment ($u_{i3}$) systems in the Jing River Basin were substituted into Equations (15)–(27) to obtain predicted means and 95% uncertainty intervals for CCD in the composite system over the study period (Figure 3).

As shown in Figure 3 and summarized in Table 3, the annual mean CCD exhibited increasing tendencies in all four subregions from 2012 to 2023, although the magnitude and statistical significance of the trends varied among subregions. The Mann–Kendall test identified statistically significant increasing trends in Pingliang and Xianyang (both p=0.016), whereas the increasing tendencies in Guyuan (p=0.086) and Qingyang (p=0.150) were not statistically significant. Despite these overall increasing tendencies, pronounced interannual fluctuations were observed across the four subregions. During 2015–2016, the CCD levels of the four subregions were at local troughs. Guyuan, Pingliang, and Qingyang reached their respective local troughs in 2015, whereas Xianyang reached its minimum in 2016. Among the four subregions, Xianyang exhibited the most pronounced decline, with its CCD uncertainty interval falling from [0.224, 0.258] in 2015 to its lowest level of [0.102, 0.138] in 2016. After 2016, the CCD recovered (to varying degrees) across subregions. Xianyang showed the most pronounced recovery, with the upper and lower limits of the CCD uncertainty interval rising by as much as 297.10% and 419.61% between 2016 and 2023, respectively, reaching [0.530, 0.548] by 2023. In Guyuan and Pingliang, CCD increased from 2016 until 2019 and 2020, respectively, after which it began to decline, falling below 0.5 by 2023 in both subregions. Qingyang showed the most modest increase in CCD over the study period, with CCD remaining within the interval of [0.350, 0.516].

**Figure 4:**
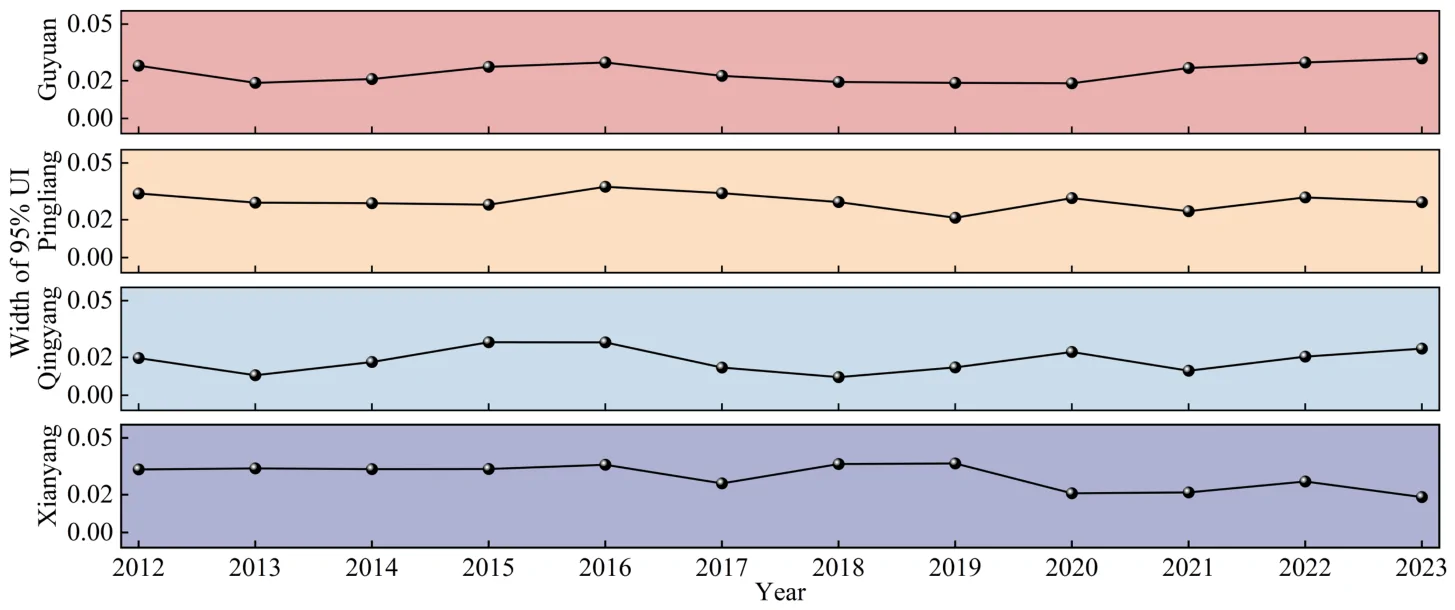
Width of the 95% uncertainty interval (UI) for coupling coordination degree (CCD) estimates of the water resources, social economy, and ecological environment (WSE) composite system in the four subregions of the Jing River Basin from 2012 to 2023.

To further examine variation in the CCD estimates, the width of the 95% uncertainty interval was calculated for each year (Figure 4). For all subregions, the width of the 95% uncertainty interval was less than 0.05, suggesting that ECCD-LMS produced CCD estimates with relatively limited dispersion despite multiple sources of uncertainty.

Figure 4 shows that the width of the 95% uncertainty interval for each subregion fluctuated to varying degrees over time, which mainly arises from the construction of uncertainty in the comprehensive evaluation connection number components and its propagation through the logical multiplication of connection numbers. As described in Step 4 (Section 2.3), Δ*a* = *ab* + *abc*, Δ*b* = *ab* + *bc*, and Δ*c* = *cb* + *cba* are used to determine the widths of the triangular fuzzy number-based dynamic value intervals for the sameness, difference, and opposition degree components, respectively. Since Δ*b* − Δ*a* = *bc*(1 − *a*) ≥ 0 and Δ*b* − Δ*c* = *ab*(1 − *c*) ≥ 0, the width of the dynamic value interval of the difference degree component is never smaller than those of the sameness and opposition degree components, indicating that the difference degree component has greater potential variability during the mutual transformations among the sameness, difference, and opposition components. In addition, the randomly sampled connection number components are normalized within each system, and their uncertainty is propagated nonlinearly through the logical multiplication of connection numbers; therefore, the width of the uncertainty interval is not determined by a simple summation of Δ*a*, Δ*b*, and Δ*c*. For a given connection number structure, larger values of Δ*a*, Δ*b*, and Δ*c* generally lead to a wider propagated uncertainty interval, whereas differences in the interval width among samples also depend on the normalized sameness–difference–opposition structure and the sensitivity of the CCD to changes in this structure.

Typical samples further verify the above characteristics. In 2016, the uncertainty interval width for Pingliang reached 0.037, the largest among all samples; the comprehensive evaluation connection numbers of both its water resources and social economy systems exhibited low sameness degrees alongside high difference degrees or opposition degrees. And the resulting estimate [0.284, 0.321] spanned the two evaluation grades of “moderate incoordination” and “mild incoordination”. Xianyang also exhibited a relatively wide interval in the same year, but its interval [0.102, 0.138] lay entirely within the grade of “severe incoordination”, indicating that the evaluation conclusion of a low coupling coordination level for this subregion was robust. By contrast, the interval width for Qingyang in 2018 was only 0.010, and its grade classification of “marginal coordination” was relatively stable. These results indicate that the mean CCD reflects the development level of the composite system, whereas the width of the 95% uncertainty interval reflects the sensitivity of the estimation results and the stability of the grade determination; together, they provide complementary information for result interpretation and decision support.

**Figure 5:**
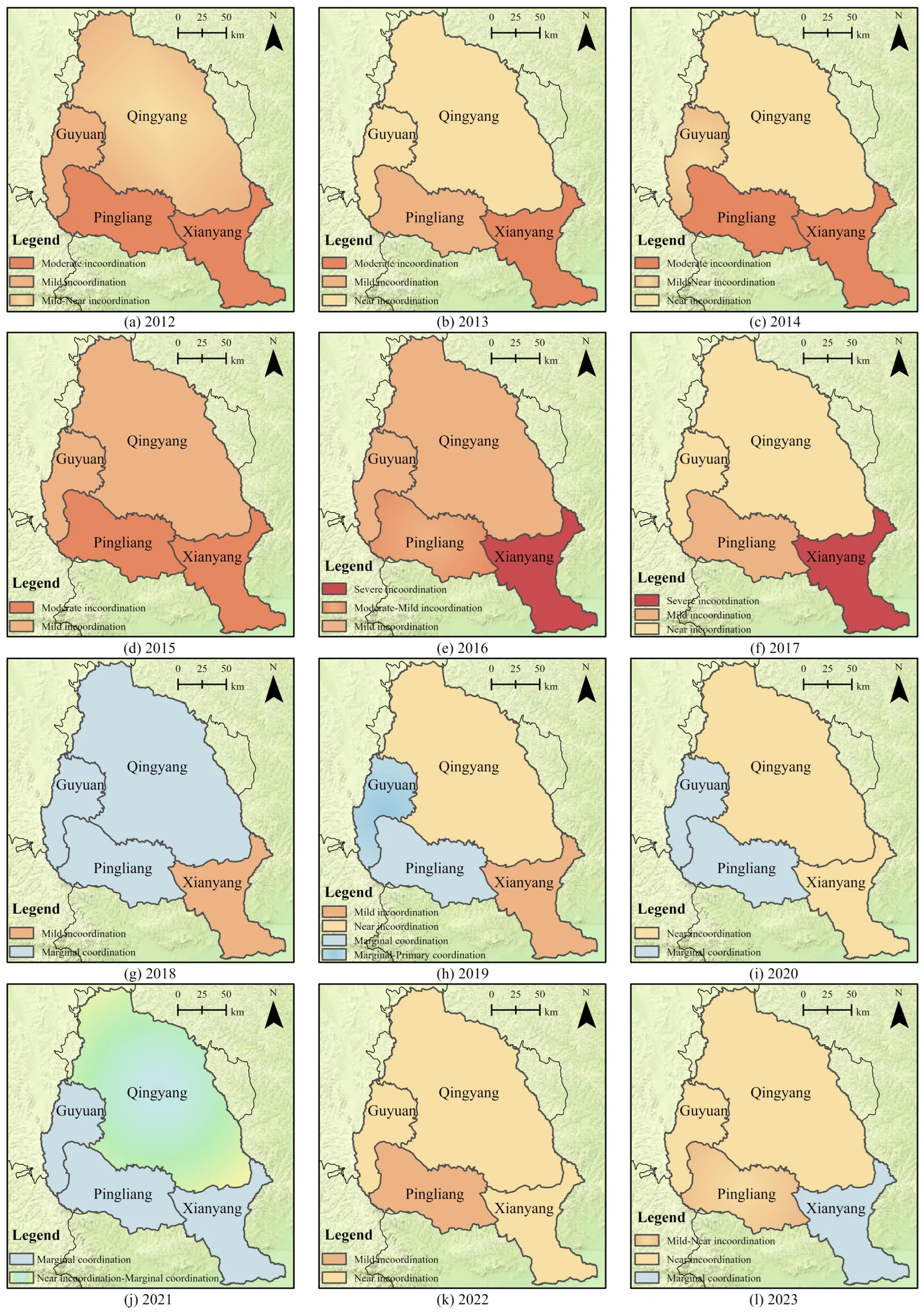
Spatiotemporal distribution of evaluation grades for the coupling coordination degree (CCD) of the water resources, social economy, and ecological environment (WSE) composite system in the four subregions of the Jing River Basin from 2012 to 2023.

Based on the grading criteria in Table 1, the corresponding evaluation grades were determined for the 95% uncertainty intervals, as shown in Figure 5. Figure 5 illustrates the spatiotemporal distribution of CCD evaluation grades in the WSE composite system of the Jing River Basin from 2012 to 2023. Over the study period, evaluation grades across focal subregions in the Jing River Basin became more similar. From 2012 to 2017, the northern and southern regions differed considerably in terms of evaluation grade. The northern region, encompassing Guyuan and Qingyang, possessed grades of “near incoordination” or “mild incoordination”, while the southern region, encompassing Pingliang and Xianyang, was graded as “moderate incoordination” or even “severe incoordination”. During this period, Xianyang consistently had the lowest evaluation grade, constraining the coordinated development of the WSE composite system. From 2018 to 2021, higher evaluation grades emerged in the western basin, with Guyuan and Pingliang classified as “marginal coordination”. However, after 2021, the evaluation grade declined in the western basin to “near incoordination” or “mild incoordination”. By 2023, only Xianyang had achieved “marginal coordination”, while the other focal subregions were mostly at “near incoordination”. Nevertheless, disparities in the evaluation grade had narrowed significantly among subregions by this point, and the overall spatial pattern had shifted from a state of highly imbalanced development to relatively balanced across the basin.

## 4. Discussion

### 4.1. Convergence Analysis for the Stochastic Simulations

**Figure 6:**
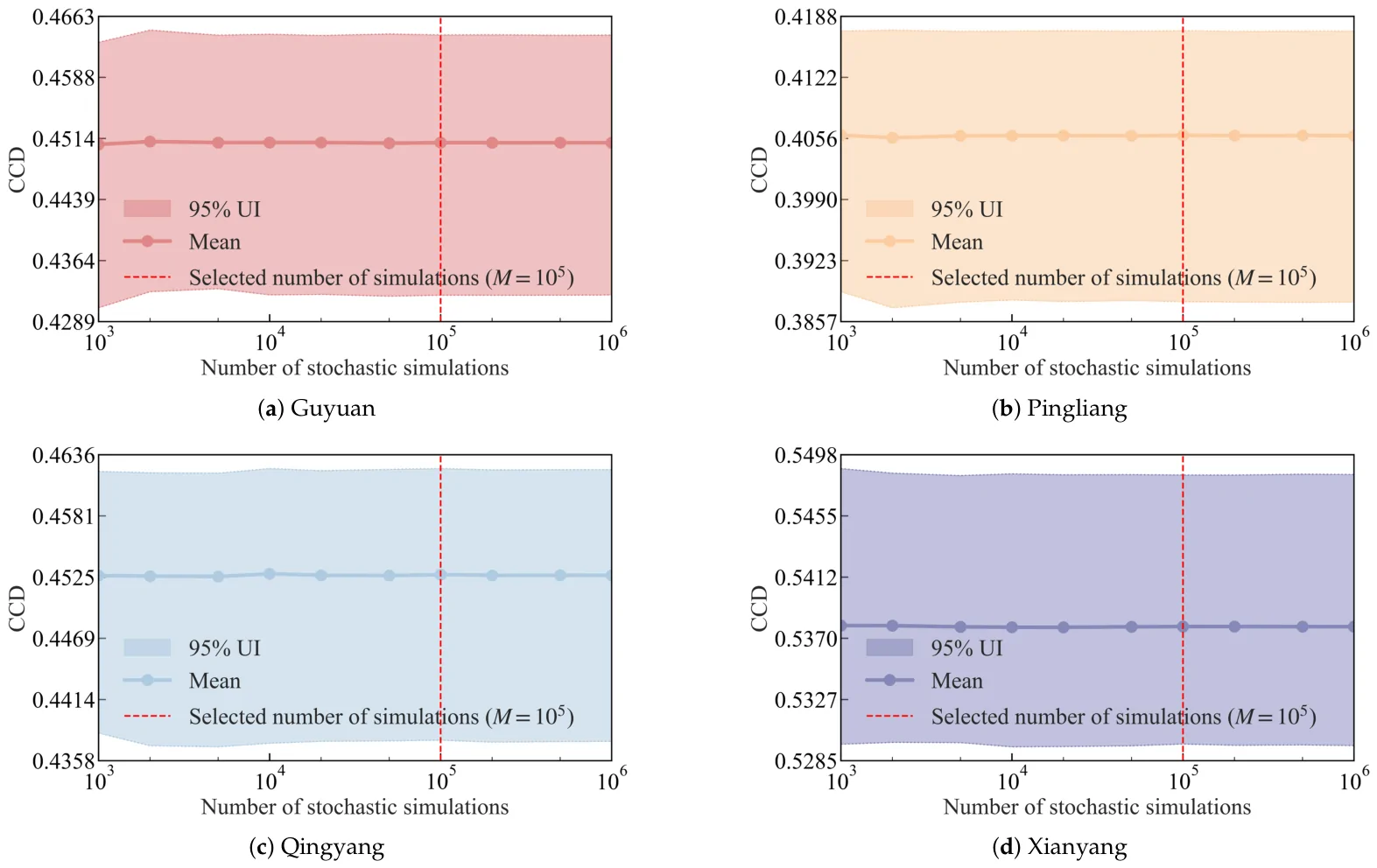
Convergence of the ECCD-LMS-derived mean coupling coordination degree (CCD) and 95% uncertainty interval (UI) as the number of stochastic simulations increased from $10^{3}$ to $10^{6}$ for the four subregions of the Jing River Basin in 2023.

When applying ECCD-LMS to determine the CCD of a composite system, the number of stochastic simulations, *M*, is a key parameter for balancing computational accuracy and operational efficiency. To determine the optimal number of simulations in this study, the convergence trajectories of the mean CCD and the upper and lower limits of the 95% uncertainty interval were assessed as *M* increased from $10^{3}$ to $10^{6}$. Due to space limitations, only the test results for 2023 are presented here (Figure 6); the convergence tests for all other years exhibit consistent convergence patterns.

When *M* < $10^{4}$ (Figure 6), the mean CCD fluctuated randomly, and the boundaries of the 95% uncertainty interval also varied with *M*; at this point, the simulation results had not yet stabilized for any of the four subregions. When $10^{4}$ ≤ *M* < $10^{5}$, the mean CCD trended toward a straight line as *M* increased, and the width of the 95% uncertainty interval also gradually stabilized. When *M* ≥ $10^{5}$, little variation was observed in the mean CCD or 95% uncertainty interval boundaries; for all subregions, the magnitudes of these variations were less than $10^{-4}$. At this point, further increasing the number of simulations to $10^{6}$ resulted in a further reduction in variation for both the mean CCD and upper and lower limits of the 95% uncertainty interval, with all estimates varying by less than $9.8\times 10^{-5}$; this suggests a very low marginal benefit to further simulations. Considering both the stability of the CCD estimates and the computational cost, the optimum number of stochastic simulations for ECCD-LMS was set to *M* = $10^{5}$. This value keeps any numerical fluctuations arising in the stochastic simulations at negligible levels, ensuring that ECCD-LMS produces stable and reproducible estimates for the study composite system.

All computations were performed in Python 3.12.7 using Spyder 6.1.1 on a computer equipped with an Intel Core i7-10750H CPU at 2.60 GHz and 32 GB of RAM. With *M* = $10^{5}$, completing the stochastic simulations for all 48 evaluation samples required an average of approximately 6.6 s over three repeated runs. This computational cost is relatively low, indicating that ECCD-LMS is feasible for practical applications.

### 4.2. Improvement of the CCD Evaluation Method

#### 4.2.1. Comparative Analysis of CCD Evaluation Methods Using Stochastic Simulations

**Figure 7:**
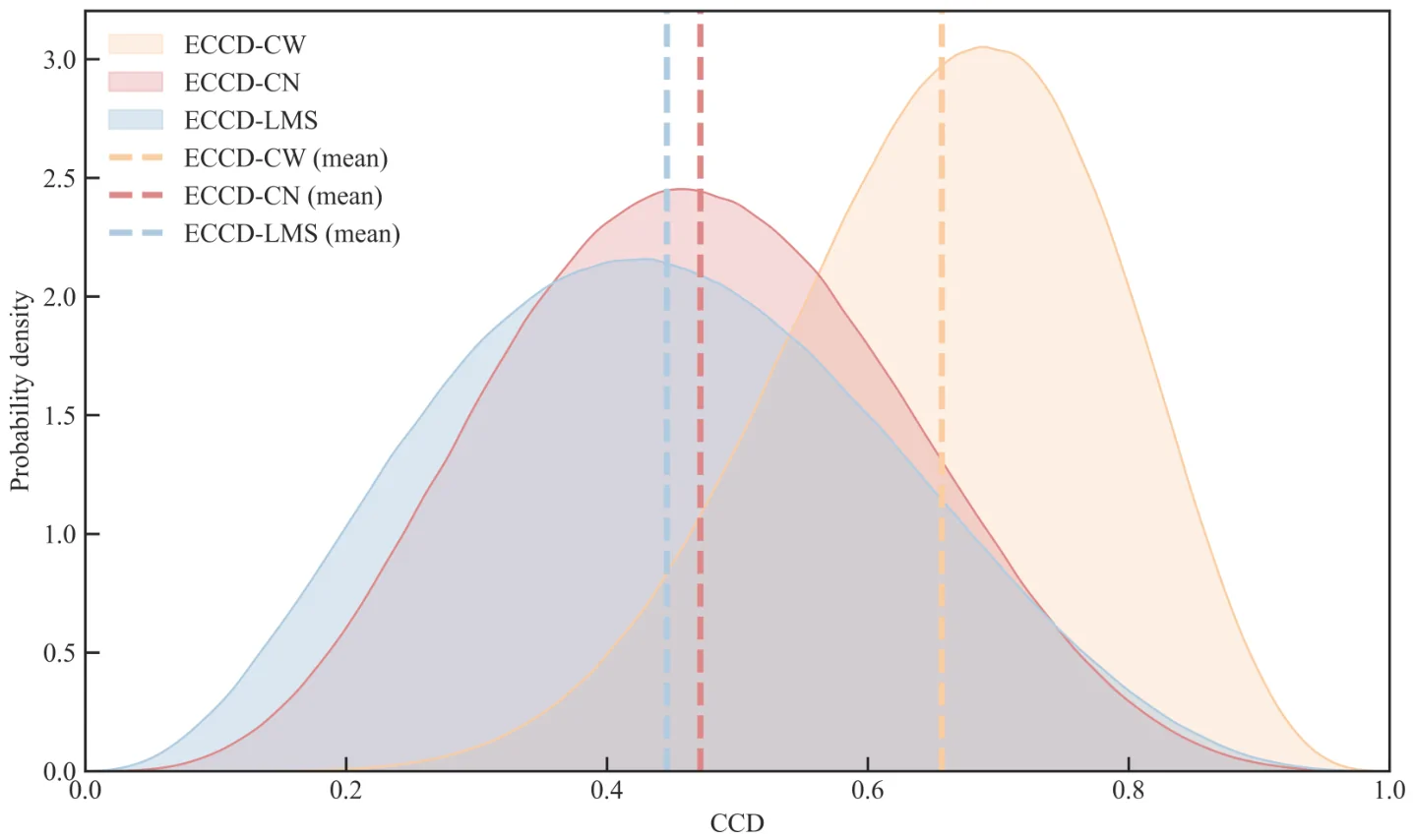
Probability density distributions of coupling coordination degree (CCD) estimates obtained using ECCD-LMS, ECCD-CN, and ECCD-CW based on stochastic simulations. The dashed vertical lines indicate the mean CCD estimates for ECCD-LMS (0.446), ECCD-CN (0.472), and ECCD-CW (0.657).

**Figure 8:**
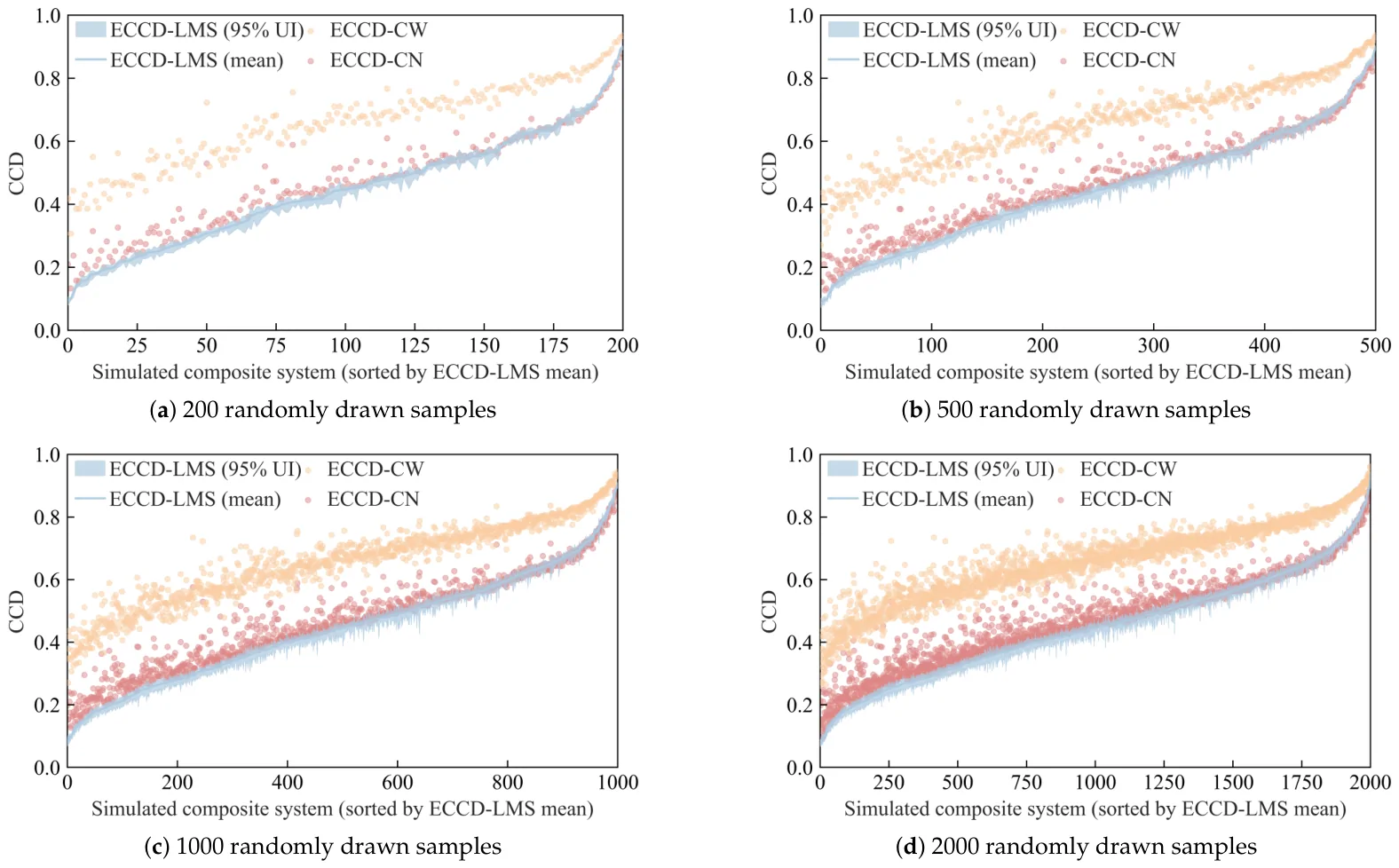
Comparison of coupling coordination degree (CCD) estimates obtained using ECCD-LMS, ECCD-CN, and ECCD-CW for randomly drawn samples with sizes of 200, 500, 1000, and 2000. The samples are ordered according to the mean CCD estimates obtained using ECCD-LMS.

To further assess the performance of ECCD-LMS in estimating the CCD of the WSE composite system, a comparative analysis based on the Monte Carlo method was conducted in this study. For each system, two independent random numbers were generated from a uniform distribution over [0, 1] and sorted in ascending order as $r_{ij}^{\left(1\right)}$ and $r_{ij}^{\left(2\right)}$. The sameness, difference, and opposition components of the comprehensive evaluation connection number were then constructed as $a_{ij}=r_{ij}^{\left(1\right)}$, $b_{ij}=r_{ij}^{\left(2\right)}-r_{ij}^{\left(1\right)}$, and $c_{ij}=1-r_{ij}^{\left(2\right)}$, respectively, thereby ensuring $a_{ij}+b_{ij}+c_{ij}=1$. The comprehensive evaluation connection numbers for the three systems (i.e., $u_{i1}$, $u_{i2}$, and $u_{i3}$) were independently generated using the same procedure. A total sample set of $N_{\mathrm{full}}=2\times 10^{6}$ virtual composite systems was constructed, covering all evaluation grades from “extreme incoordination” to “excellent coordination”. Each sample contained a set of $u_{i1}$, $u_{i2}$, and $u_{i3}$. To compare CCD evaluation methods, all sample set data were substituted into ECCD-CW [8], ECCD-CN [14], and ECCD-LMS methods, and probability density plots were created for CCD estimates from each method (see Figure 7 and Table 4 for key method characteristics). To ensure comparability between ECCD-LMS, which outputs both means and uncertainty intervals, and the other methods, the ECCD-LMS mean CCD estimates were used to plot the probability density distribution. Next, to assess the stability of ECCD-LMS across sample sizes and to compare the CCD distribution among methods, subsamples of $N_{200}=200$, $N_{500}=500$, $N_{1000}=1000$, and $N_{2000}=2000$ were randomly drawn from the total sample set. The resulting CCD estimates were sorted in ascending order based on the ECCD-LMS values, and these results were plotted separately for each sample size (Figure 8). The ECCD-LMS method results were compared to those of ECCD-CN and ECCD-CW based on four criteria: CCD distribution characteristics, evaluation objectivity, result stability, and uncertainty quantification (Figure 7 and Figure 8; Table 4).

As can be seen from Figure 7, the distribution of CCD values from ECCD-CW was strongly left-skewed, with its centroid notably shifted toward higher CCD values (mean CCD = 0.657). This suggests a tendency for ECCD-CW to overestimate CCD in the evaluation process, meaning that, given identical input data, ECCD-CW tended to produce systematically higher CCD estimates than the other methods. The probability density curves for ECCD-LMS and ECCD-CN followed roughly normal distributions, with means of 0.446 and 0.472, respectively. Thus, ECCD-LMS and ECCD-CN produced more objective and neutral CCD estimates from completely random input data, without exhibiting the systematic upward shift observed for ECCD-CW.

The standard deviation reflects the dispersion of CCD estimates produced by each evaluation method. The standard deviation was 0.169 for ECCD-LMS (Table 4), higher than that for ECCD-CN (0.152) and ECCD-CW (0.127), indicating greater dispersion in the CCD estimates. Together with its wider overall numerical range, this result shows that ECCD-LMS produces a broader spread of CCD values among the randomly generated composite systems, allowing differences among evaluation samples to be more clearly reflected in the evaluation results.

As the number of subsamples increased from 200 to 2000 (Figure 8a–d), the distribution of CCD estimates remained relatively consistent across the three evaluation methods. Regardless of the sample size, CCD values from ECCD-CW were consistently higher than those from ECCD-CN and ECCD-LMS, confirming the tendency of ECCD-CW to systematically overestimate CCD. Meanwhile, CCD estimates from ECCD-CN varied around the ECCD-LMS curve. At lower CCD values, ECCD-CN estimates were generally higher than the ECCD-LMS mean, while the opposite was true at higher CCD values. This indicates that ECCD-CN estimates were relatively more concentrated toward the middle of the [0, 1] interval, whereas ECCD-LMS produced a broader numerical spread across samples. The distribution of ECCD-LMS CCD estimates (means and 95% uncertainty intervals) was similar across sample sizes. This consistency (also seen in the curves for ECCD-CN and ECCD-CW) suggests that CCD estimation was not affected by the sample size.

Compared to ECCD-CW and ECCD-CN, which are limited to providing static point estimates, ECCD-LMS further characterizes the uncertainty associated with CCD estimates by constructing uncertainty intervals. The blue shaded areas in Figure 8a–d display the 95% uncertainty intervals obtained from ECCD-LMS. Across sample sizes, the width of the uncertainty interval was not constant but instead varied among samples, depending on shifts in the comprehensive evaluation connection number components for the water resources, social economy, and ecological environment systems. With the aid of the output uncertainty intervals, even at sample points where the CCD mean is close to estimated values from ECCD-CN, ECCD-LMS further quantifies the magnitude of uncertainty through variations in uncertainty interval width. This characteristic enables ECCD-LMS to provide additional information on uncertainty while evaluating the CCD of regional WSE composite systems, thereby providing more comprehensive support for the formulation of regulatory strategies.

In summary, method comparisons revealed that ECCD-LMS avoided the systematic upward tendency observed for ECCD-CW while maintaining the evaluation objectivity of ECCD-CN; in addition, it produced a broader numerical spread of CCD estimates across samples. More importantly, by utilizing stochastic simulations, the ECCD-LMS method effectively addressed problems with traditional evaluation methods related to uncertainty quantification. Whether in small-sample or large-sample settings, ECCD-LMS demonstrated excellent stability, providing a more informative tool for the scientific evaluation of the CCD in regional WSE composite systems.

#### 4.2.2. Comparative Analysis of Evaluation Methods Based on a Practical Case

**Figure 9:**
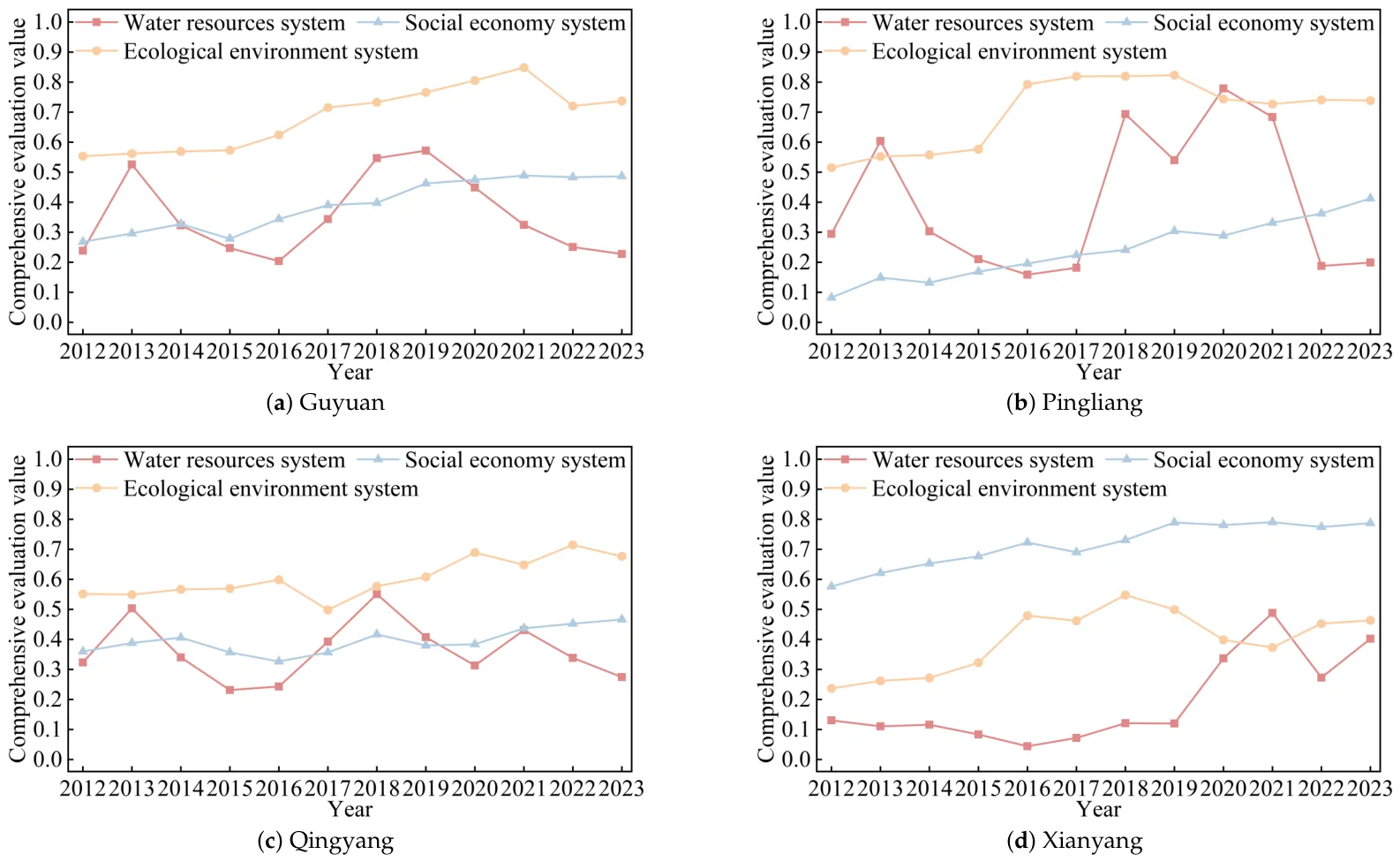
Comprehensive evaluation values of the water resources, social economy, and ecological environment systems for the four subregions of the Jing River Basin from 2012 to 2023.

**Figure 10:**
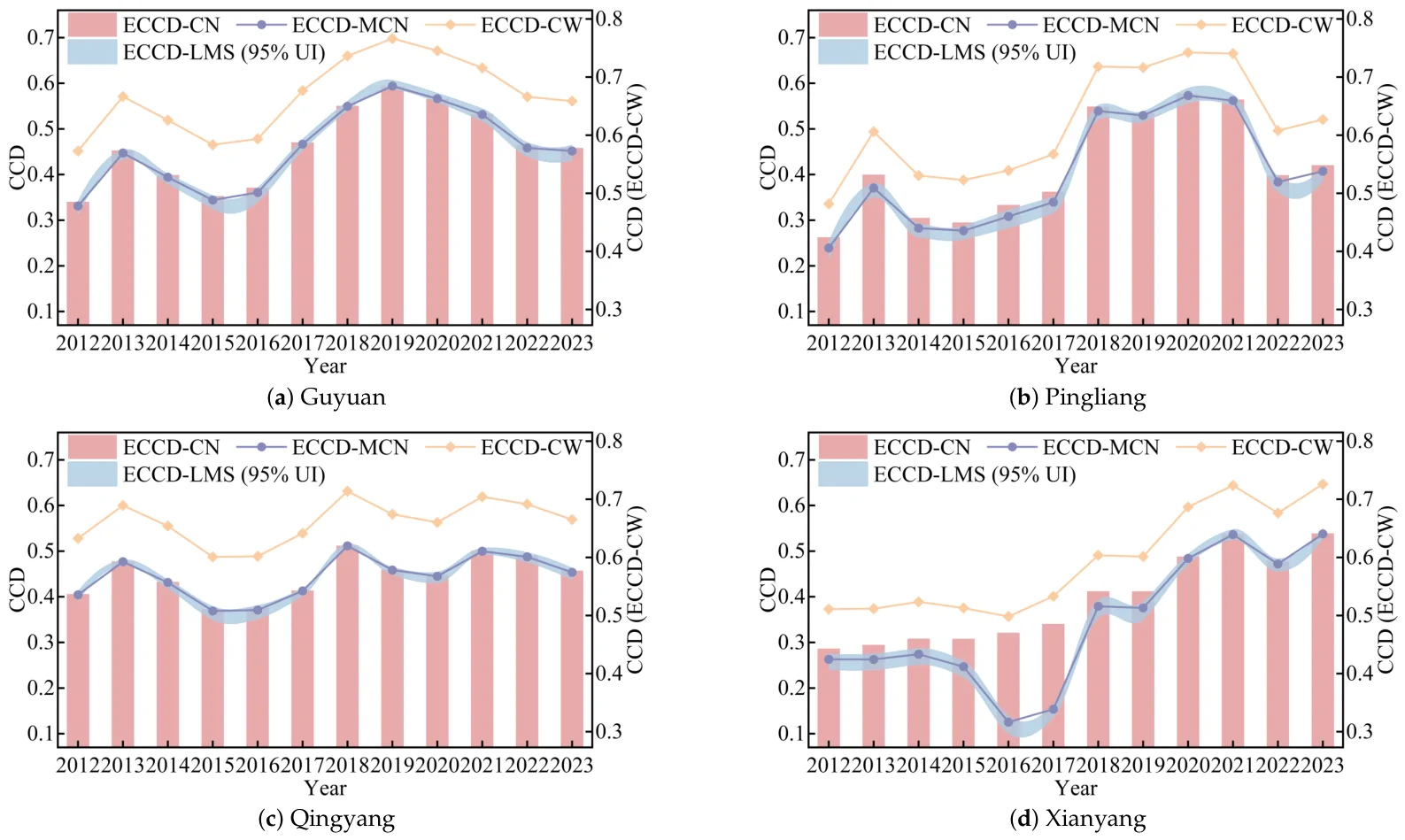
Comparison of the coupling coordination degree (CCD) estimates obtained using ECCD-LMS, ECCD-MCN, ECCD-CN, and ECCD-CW for the four subregions of the Jing River Basin from 2012 to 2023.

To further compare CCD evaluation methods, data of the 20 evaluation indicators for each subregion in the Jing River Basin were substituted into ECCD-CW [8], ECCD-CN [14], and ECCD-MCN [15]. The results from these methods, including comprehensive evaluation values and CCD estimates, were then compared with those of ECCD-LMS, as shown in Figure 9 and Figure 10.

In the stochastic simulation-based method comparison (Section 4.2.1), ECCD-CW produced systematically higher CCD estimates than the other methods. This characteristic was further observed in the practical case of the Jing River Basin, where the CCD estimates from ECCD-CW were consistently higher than those from the other methods (Figure 10). This characteristic can be explained by the mathematical formulation of ECCD-CW. For sample *i*, let $G_{i}={\left(U_{i1}U_{i2}U_{i3}\right)}^{1/3}$ and $A_{i}=\left(U_{i1}+U_{i2}+U_{i3}\right)/3$ denote, respectively, the geometric and arithmetic means of the comprehensive evaluation values of the three systems. The coupling degree calculated by ECCD-CW is $C_{i}=G_{i}/A_{i}$. When the comprehensive evaluation values of the three systems are relatively close, $G_{i}$ and $A_{i}$ are likewise close, and $C_{i}$ therefore approaches 1. Under equal weighting of the three systems, the comprehensive evaluation value of the composite system becomes $T_{i}=A_{i}$, and thus $D_{i}={\left(C_{i}T_{i}\right)}^{1/2}=G_{i}^{1/2}$. Since the comprehensive evaluation values of the individual systems all lie within [0,1], $G_{i}^{1/2}>G_{i}$ whenever $0<G_{i}<1$. This indicates that the mathematical formulation of ECCD-CW tends to produce larger CCD estimates.

For example, the comprehensive evaluation values of the water resources, social economy, and ecological environment systems for Qingyang in 2013 were 0.503, 0.388, and 0.549, respectively. Accordingly, $A_{i}=\left(0.503+0.549+0.388\right)/3=0.480$ and $G_{i}={\left(0.503\times 0.549\times 0.388\right)}^{1/3}=0.475$, giving $C_{i}=G_{i}/A_{i}=0.990$. Under equal weighting, $T_{i}=A_{i}=0.480$, and thus $D_{i}={\left(C_{i}T_{i}\right)}^{1/2}=G_{i}^{1/2}=0.689$. The relatively high coupling degree resulted mainly from the closeness of the geometric and arithmetic means of the three system evaluation values. The subsequent square-root operation resulted in a CCD value of 0.689. This practical calculation further illustrates why ECCD-CW tends to produce systematically higher CCD estimates.

By contrast to ECCD-CW, ECCD-LMS calculates the CCD through the logical multiplication of three-element connection numbers for each system, which better reflects the importance of the comprehensive evaluation level of each system for the CCD of the composite system. That is, as long as any system has a relatively low comprehensive evaluation level, it will lead to a low CCD value [8]. For example, in 2016, the annual total precipitation, per capita water resources, and water yield modulus, within Xianyang were all at their lowest values of the study period, being $30.11\times 10^{8}$ m^3^, 77.00 m^3^/person, and $3.79\times 10^{4}$ m^3^/km^2^, respectively; this led the comprehensive evaluation value of the water resources system to decrease from 0.083 to 0.044. Under particularly unfavorable water-resource conditions, the mean CCD value for ECCD-LMS dropped to 0.124, which demonstrates the method’s sensitivity and ability to capture the effects of constraints from individual systems on the coordinated development of the composite system. In contrast, the CCD value for ECCD-CN, calculated based on a geometric mean, was much higher (0.321), likely due to the influence of higher comprehensive evaluation values for the social economy and ecological environment systems. Meanwhile, the CCD value for ECCD-CW, despite showing a declining trend, remained very high at 0.498, constrained by the irrationality of the underlying method calculations.

The numerical spread of CCD estimates reflects the extent to which an evaluation method represents differences among samples at different developmental stages. Comparing the evaluation methods for the Jing River Basin dataset, the mean CCD values from ECCD-LMS ranged from 0.124–0.595, a wider range than that for ECCD-CW (0.482–0.766) or ECCD-CN (0.263–0.594), indicating a broader numerical spread of the evaluation results. Accordingly, ECCD-LMS more clearly reflected differences in the coupling coordination status of the WSE composite system across subregions and years in the Jing River Basin.

Compared with the static point estimates of ECCD-MCN, the ECCD-LMS method outputs evaluation results that include a 95% uncertainty interval, thereby addressing the limitation of traditional methods in characterizing uncertainty in the evaluation results. As shown in Figure 4 and Figure 10, in the practical case study, the 95% uncertainty intervals obtained using ECCD-LMS fully encompassed the corresponding estimates from ECCD-MCN, and their widths were consistently less than 0.05, suggesting relatively limited propagated uncertainty in the ECCD-LMS estimates. As discussed in Section 3.3, the width of the uncertainty interval is jointly shaped by the component variation ranges determined by Δa, Δb, and Δc, and their nonlinear propagation through the logical multiplication of connection numbers. The mathematical properties of Δa, Δb, and Δc further help explain the differences in interval width among samples. From Δb=b(a+c)=b(1−b), Δb reaches its maximum value when b=0.5 and approaches zero when *b* approaches zero or one. From Δa=ab+abc=ab(1+c), for a given value of *c*, Δa reaches its maximum value when a=b=(1−c)/2 and approaches zero when *a* or *b* approaches zero. Similarly, because Δc=cb+cba=cb(1+a) is structurally symmetric to Δa, for a given value of *a*, Δc reaches its maximum value when c=b=(1−a)/2 and approaches zero when *c* or *b* approaches zero. These properties indicate that different sameness–difference–opposition structures generate different potential ranges of component variation. In general, when the resulting values of Δa, Δb, and Δc are collectively smaller, the propagated uncertainty interval tends to be narrower, whereas larger component variation generally increases the potential for a wider interval; the final interval width, however, also depends on the normalized connection number structure and its nonlinear propagation through the logical multiplication of connection numbers.

### 4.3. Associations Between Comprehensive Evaluation Values of Individual Systems and CCD

The comprehensive evaluation values of the social economy and ecological environment systems were relatively stable over time (Figure 9), while those of the water resources system exhibited larger fluctuations, reflecting greater variability in natural conditions. Comparing Figure 9 and Figure 10, the comprehensive evaluation values of the water resources system showed temporal variations similar to those of the mean CCD estimates of the composite system. To quantitatively examine the associations between these temporal variations, grey relational analysis [42] was conducted separately for each subregion. The mean CCD estimates obtained by ECCD-LMS were taken as the reference sequence $X_{0}\left(t\right)$, while the comprehensive evaluation values of the water resources, social economy, and ecological environment systems were used as the comparison sequences $X_{s}\left(t\right)$. The absolute difference was calculated as $\Delta_{s}\left(t\right)=\left|X_{0}\left(t\right)-X_{s}\left(t\right)\right|$, and the grey relational coefficient was determined as $\xi_{s}\left(t\right)=\left(\Delta_{\min}+\rho \Delta_{\max}\right)/\left[\Delta_{s}\left(t\right)+\rho \Delta_{\max}\right]$. The grey relational degree was then obtained as $\gamma_{s}=\left(1/12\right)\sum_{t=1}^{12}\xi_{s}\left(t\right)$. Here, *t* denotes the 12 study years from 2012 to 2023, and *s* denotes the water resources, social economy, or ecological environment system; $\Delta_{\min}$ and $\Delta_{\max}$ are the minimum and maximum absolute differences, respectively, among the three comparison sequences over the 12 years within each subregion; and ρ is the distinguishing coefficient, set to 0.5 in this study. The mean grey relational degree for the Jing River Basin was obtained by averaging the grey relational degrees of the four subregions.

The mean grey relational degrees between the comprehensive evaluation values of the water resources, social economy, and ecological environment systems and the mean CCD estimates were 0.666, 0.653, and 0.541, respectively. The water resources system exhibited the highest mean grey relational degree with CCD among the three systems, although it was only slightly higher than that of the social economy system. This temporal association can also be observed in representative years. For example, the large increase in the comprehensive evaluation value of the water resources system in Pingliang from 2017 to 2018 coincided with a notable rise in the CCD of the composite system, while a similar decline from 2021 to 2022 was accompanied by a synchronous decrease in the CCD. In Xianyang, the comprehensive evaluation value of the water resources system dropped to its lowest point in 2016, and the minimum CCD value also occurred in 2016. Taken together, these results suggest that year-to-year variations in the comprehensive evaluation level of the water resources system were closely associated with variations in the CCD of the WSE composite system.

## 5. Conclusions

The evaluation of coupling coordination degree (CCD) in regional water resources, social economy and ecological environment (WSE) composite system aims to comprehensively assess the coordinated development of these systems, and CCD evaluation methods represent commonly used approaches to estimate CCD. However, traditional methods fail to ensure CCD estimates are both accurate and reliable and to quantify uncertainty in these estimates. To address these deficits, this study advanced a novel CCD evaluation method, ECCD-LMS, that incorporates overall partial connection numbers, triangular fuzzy numbers, and Monte Carlo simulations. The ECCD-LMS method estimates the CCD of regional WSE composite systems by combining the logical multiplication of connection numbers with stochastic simulations. To demonstrate the method, an empirical study was conducted in the Jing River Basin. The main conclusions are described below.

(1) Together, method comparisons based on random, simulated data and a practical case study indicate that ECCD-LMS can effectively characterize how each system’s state conforms to the overall development objectives of the composite system. The proposed method not only effectively corrected the systematic overestimation associated with ECCD-CW but also produced a broader numerical spread of CCD estimates that more clearly reflected differences among evaluation samples, while maintaining objectivity comparable to that of ECCD-CN. Meanwhile, ECCD-LMS provides both mean CCD estimates and associated 95% uncertainty intervals, overcoming a key limitation of traditional methods that only provide static point estimates.

(2) By assessing method convergence through stochastic simulations and comparing method performance across sample sizes, the ECCD-LMS method was found to demonstrate good stability. When the number of stochastic simulations (*M*) equaled $10^{5}$, variation in the CCD estimates was less than $10^{-4}$. Across sample sizes, the distribution of the ECCD-LMS CCD estimates remained consistent, indicating that method outputs did not depend on the choice of sample size. Furthermore, the width of the 95% uncertainty interval in the practical case study (based on real-world data) remained below 0.05, indicating relatively limited propagated uncertainty in the CCD estimates under the combined influence of multiple sources of uncertainty.

(3) Applying the ECCD-LMS method to the Jing River Basin, the annual mean CCD exhibited increasing tendencies in all four subregions from 2012 to 2023, among which Pingliang and Xianyang showed significant upward trends. However, year-to-year fluctuations in CCD were closely associated with variations in the comprehensive evaluation level of the water resources system. The spatial distribution of the evaluation grades revealed pronounced disparities among subregions in the Jing River Basin early in the study period, with subregions becoming more similar in evaluation grade over time. The ECCD-LMS method accurately captured the sharp decline in CCD observed for Xianyang in 2016 under particularly unfavorable water-resource conditions, illustrating the method’s sensitivity to changes in the water resources system and the physical interpretability of the evaluation results.

In summary, the ECCD-LMS method proposed here combines set pair reasoning, set pair analysis, and stochastic simulations to quantify uncertainty in the CCD evaluation process of WSE composite systems, highlighting the application potential of set pair logical reasoning. However, as an indicator-based comprehensive evaluation method, ECCD-LMS remains dependent on the construction of the evaluation index system, grading criteria, and indicator weights; these inputs should therefore be appropriately adapted and validated when the method is applied to different regions or types of composite systems. In addition, triangular fuzzy numbers were adopted in this study to characterize stochastic variations in the connection number components, and alternative probability distributions or fuzzy structures should be further examined for uncertainty propagation. Although the present case study demonstrated a relatively low computational cost, the computational performance of ECCD-LMS in larger-scale composite systems involving more component systems, evaluation indicators, and samples requires further assessment. Future work may expand ECCD-LMS applications to obstacle factor diagnosis and scenario simulations, such as land-use change and water resources allocation scenarios, to broaden its application in regional WSE management.

## Figures and Tables

**Table 1:** Coupling coordination degree (CCD) grading criteria for the regional WSE composite system.

Development Stage	CCD	Evaluation (i.e., Coupling Coordination) Grade
Imbalanced development stage	[0.00, 0.10]	$C_{1}$, Extreme incoordination
	(0.10, 0.20]	$C_{2}$, Severe incoordination
	(0.20, 0.30]	$C_{3}$, Moderate incoordination
	(0.30, 0.40]	$C_{4}$, Mild incoordination
Transitional development stage	(0.40, 0.50]	$C_{5}$, Near incoordination
	(0.50, 0.60]	$C_{6}$, Marginal coordination
Balanced development stage	(0.60, 0.70]	$C_{7}$, Primary coordination
	(0.70, 0.80]	$C_{8}$, Intermediate coordination
	(0.80, 0.90]	$C_{9}$, Good coordination
	(0.90, 1.00]	$C_{10}$, Excellent coordination

**Table 2:** Index system, grading criteria, and weights for evaluating the CCD of the Jing River Basin WSE composite system.

System	Indicator	Unit	Evaluation Grades			Weight	Indicator Type (+/−)
			Grade I (Coordinated)	Grade II (Marginally Coordinated)	Grade III (Uncoordinated)		
Water resources	$W_{1}$, Per capita water resources	m^3^/person	>500	[300, 500]	<300	0.292	+
	$W_{2}$, Water yield modulus	$10^{4}$ m^3^/km^2^	>10	[5, 10]	<5	0.380	+
	$W_{3}$, Annual total precipitation	$10^{8}$ m^3^	>140	[75, 140]	<75	0.213	+
	$W_{4}$, Water supply modulus	$10^{4}$ m^3^/km^2^	<5	[5, 10]	>10	0.115	−
Social economy	$S_{1}$, Urbanization rate	%	>45	[30, 45]	<30	0.055	+
	$S_{2}$, Proportion of the tertiary industry	%	>55	[35, 55]	<35	0.071	+
	$S_{3}$, Per capita GDP	$10^{4}$ CNY/capita	>5	[2.5, 5]	<2.5	0.160	+
	$S_{4}$, Population growth rate	‰	>7	[2, 7]	<2	0.053	+
	$S_{5}$, Population density	persons/km^2^	>350	[200, 350]	<200	0.270	+
	$S_{6}$, Water consumption per 10^4^ CNY of GDP	m^3^	<35	[35, 75]	>75	0.167	−
	$S_{7}$, Farmland irrigation water consumption per mu	m^3^	<100	[100, 250]	>250	0.098	−
	$S_{8}$, Water consumption per $10^{4}$ CNY of industrial value-added	m^3^	<15	[15, 45]	>45	0.126	−
Ecological environment	$E_{1}$, Forest cover	%	>30	[10, 30]	<10	0.233	+
	$E_{2}$, Per capita green area	m^2^/capita	>30	[20, 30]	<20	0.075	+
	$E_{3}$, Percent cover of greenery in built-up areas	%	>40	[30, 40]	<30	0.089	+
	$E_{4}$, Ecological water use rate	%	>5	[2, 5]	<2	0.060	+
	$E_{5}$, Annual total wastewater discharge	$10^{4}$ t	<2000	[2000, 6000]	>6000	0.210	−
	$E_{6}$, COD discharge in wastewater	$10^{4}$ t	<0.5	[0.5, 3]	>3	0.177	−
	$E_{7}$, Ammonia nitrogen discharge in wastewater	t	<800	[800, 2300]	>2300	0.115	−
	$E_{8}$, Water consumption rate	%	<60	[60, 70]	>70	0.041	−

Note: “+” and “−” denote positive and negative indicators, respectively. CNY, Chinese Yuan, the official currency of China; COD, chemical oxygen demand; mu, a Chinese unit of land area (1 mu ≈ 666.67 m^2^).

**Table 3:** Mann–Kendall trend test results and Sen’s slope estimates for the annual mean coupling coordination degree (CCD) in four subregions of the Jing River Basin from 2012 to 2023.

Subregion	*Z*	*p*	Sen’s Slope	Trend
Guyuan	1.714	0.086	0.0150	Increasing, not significant
Pingliang	2.400	0.016	0.0180	Increasing, significant
Qingyang	1.440	0.150	0.0069	Increasing, not significant
Xianyang	2.400	0.016	0.0293	Increasing, significant

Note: The two-sided Mann–Kendall test was applied separately to the annual mean CCD series of each subregion. Sen’s slope represents the annual rate of change in CCD, and trends were considered statistically significant at p<0.05.

**Table 4:** Statistical overview of the coupling coordination degree (CCD) estimates from ECCD-LMS, ECCD-CN, and ECCD-CW based on stochastic simulations.

Statistical Characteristic	ECCD-LMS	ECCD-CN	ECCD-CW
Sample size	$2\times 10^{6}$	$2\times 10^{6}$	$2\times 10^{6}$
Mean	0.446	0.472	0.657
Standard deviation	0.169	0.152	0.127
Minimum	0.004	0.017	0.093
$Q_{5}$	0.178	0.227	0.433
$Q_{25}$	0.320	0.362	0.573
$Q_{50}$	0.440	0.469	0.666
$Q_{75}$	0.566	0.579	0.750
$Q_{95}$	0.736	0.727	0.849
Maximum	0.985	0.983	0.992

Note: *Q*_5_, *Q*_25_, *Q*_50_, *Q*_75_, and *Q*_95_ represent the 5th, 25th, 50th, 75th, and 95th percentiles, respectively.

## Data Availability

The data used in this study were compiled from the official statistical yearbooks, water resources bulletins, and statistical communiqués described in Section 2.2. Owing to data management considerations associated with the source materials, the compiled dataset is not publicly available.
